# An Affordable Wet Chemical Route to Grow Conducting Hybrid Graphite-Diamond Nanowires: Demonstration by A Single Nanowire Device

**DOI:** 10.1038/s41598-017-11741-9

**Published:** 2017-09-11

**Authors:** Muthaiah Shellaiah, Tin Hao Chen, Turibius Simon, Liang-Chen Li, Kien Wen Sun, Fu-Hsiang Ko

**Affiliations:** 1 Department of Applied Chemistry, National Chiao Tung University, Hsinchu, 300 Taiwan; 2 Department of Materials Science and Engineering, National Chiao Tung University, Hsinchu, 300 Taiwan; 3 Center for Nano Science and Technology, National Chiao Tung University, Hsinchu, 300 Taiwan; 4 Department of Electronics Engineering, National Chiao Tung University, Hsinchu, 300 Taiwan

## Abstract

We report an affordable wet chemical route for the reproducible hybrid graphite-diamond nanowires (G-DNWs) growth from cysteamine functionalized diamond nanoparticles (**ND-Cys**) via pH induced self-assembly, which has been visualized through SEM and TEM images. Interestingly, the mechanistic aspects behind that self-assembly directed G-DNWs formation was discussed in details. Notably, above self-assembly was validated by AFM and TEM data. Further interrogations by XRD and Raman data were revealed the possible graphite sheath wrapping over DNWs. Moreover, the HR-TEM studies also verified the coexistence of less perfect sp^2^ graphite layer wrapped over the sp^3^ diamond carbon and the impurity channels as well. Very importantly, conductivity of hybrid G-DNWs was verified via fabrication of a single G-DNW. Wherein, the better conductivity of G-DNW portion L2 was found as 2.4 ± 1.92 × 10^−6^ mS/cm and revealed its effective applicability in near future. In addition to note, temperature dependent carrier transport mechanisms and activation energy calculations were reported in details in this work. Ultimately, to demonstrate the importance of our conductivity measurements, the possible mechanism behind the electrical transport and the comparative account on electrical resistivities of carbon based materials were provided.

## Introduction

Owing to the various applications^1–5^, nanowires (NWs) growth is become an attractive research field. However, in that direction the development conducting or semi conducting NWs were highly essential to apply in photonics or solar cells^6, 7^, which seems to be vital for modern world. Therefore, many research groups are currently tend to develop such conducting or semi-conducting metallic NWs^8–10^. In this way, diamond nanowires (DNWs) were also attracted consideration by its effective applications in electrochemical, sensing and semiconducting studies^11–14^. Further to note, the hybrid graphite-DNWs (G-DNWs) were already known as promising candidate in nanoelectronics^15–17^. Wherein, the insertion of sp^2^ graphene carbon on the surface of insulating diamond or metal nanowires may certainly enhance their conductivity^18, 19^. Likewise, graphite sheath wrapping on DNWs, might improve conductivity by tuning the sp^3^/sp^2^ carbon ratio^20^. But, developing such G-DNWs majorly involved the chemical vapor deposition (CVD) techniques^21–24^, which requires expensive instruments to maintain the high temperature. Hence, led the scientists to find out alternate cost effective way.

As illustrated previously, diamond nanoparticles (DNPs) may undergo electrostatic self-assembly^25–28^, which can be further tuned *via* adjusting the pH of dispersive solution. In which, similar to laser, bond-dangling, photon energy and metal ions^29–32^, the pH of the growing system may also act as a source to enhance partial graphitization of DNPs. Additionally, upon controlling the dispersive concentration of DNPs^33^, self-assembly may led to the formation of hybrid G-DNWs. For instance, Shang *et al*. reported such a self-assembled growth of ultrathin diamond nanorods with 2.1 nm diameter applied in field emission studies^34^. Nevertheless, they have also utilize microwave plasma chemical vapour deposition (MPCVD) method to grow those nanorods from commercial diamond cluster. Instead, Kuang and Xie *et al*., reported a pH induced synthesis and self-assembly of 3D layered β-FeOOH nanorods^35^. Hence, we protracted our vision towards pH induced self-assembly of DNPs to grow G-DNWs.

Next, to initiate the effective self-assembly by tuning the pH of growing solution, the functional group introduction on the surface of DNPs might be considered, which may also increase the charge transfer ability of G-DNWs^36^. On this way, Sheu, *et al*., discussed about the electron transport ability of cysteamine in a single-polypeptide transistor studies^37^. Additionally, doping of S and N atoms on the graphitized nanodiamond surface were also boosted its electrocatalytic activity^29^. Therefore, to improve the novelty and electrical transport properties of formed G-DNWs, we tend to functionalize our DNPs by Cysteamine (containing S and N atoms) *via* wet chemical synthesize. Next, due to the complications of single nanowire fabrication, so far single G-DNW has not been fabricated towards conductivity studies. However, as demonstrated by our previous reports on thermoelectric studies on Sb_2_Se_3_ and poly(3-hexylthiophene) single nanowires^38, 39^, Very excitingly, in this paper we successfully report the conductivity study of fabricated single G-DNW.

Herein, we report the pH induced self-assembly of novel cysteamine functionalized diamond nanoparticles (**ND-Cys**) to evidence hybrid G-DNWs growth. The formed G-DNWs were well authenticated by conductivity and transport mechanistic studies of a chosen single G-DNW.

## Methods

### General Information

Industrial nanodiamond powder with 4–12 nm sized was commercially purchased from BAOO-WEI INTERNATIONAL CO., LTD., Taiwan. 2-aminoethanethiol (commercially known as cysteamine) and Thionyl chloride (SOCl_2_) were purchased from Sigma Aldrich. All anhydrous reactions were carried out by standard procedures under nitrogen atmosphere to avoid moisture. The solvents were dried by distillation over appropriate drying agents. Identification and purity of ND-Cysteamine **(ND-Cys)** was characterized by Fourier transform Infrared spectroscopy (FTIR), Raman spectroscopy, Zeta potential and Energy Dispersive Spectrum (EDX) studies. SEM and EDX studies were carried out by JEOL-JSM-6700. TEM and HR-TEM studies were done by JEOL-JEM-2100 and JEOL-JEM-2100F, respectively. Further, AFM studies were performed by tapping mode D3100. The size distribution and zeta potential were obtained from dynamic light scattering BECKMAN COULTER Delsa^TM^ Nano C particle analyzer. FTIR investigations were proceeded by Perkin Elmer - 100 FT-IR SPECTRUM ONE spectrometer. Raman interrogations were employed by HOROBA, Lab RAM HR instrumental set up and adopted the DPSS 488 nm laser. The powder XRD data of ND-p, NDA and **ND-Cys** were obtained from BRUKER AXS D2 Phaser (a26-x1-A2BOE2B).

Fabrication of a single diamond nanowire (DNW) involved the following instruments. The Plasma-Enhanced Chemical Vapor Depositionn of SiO_2_ (300 nm) over N-type Si substrate was done by OXFORD INSTRUMENTS, Plasmalab80Plus. Further, the pad was completed through Mask Alignment and Exposure System (Model 60 DUV/MUV/NearUV). Then, alignment mark and completion of elctrode connection has been done by E-beam Lithography System (ELS - 7500EX). Next, to fixed nanowire and improved contact, Focus Ion Beam (model: FEI Nova 200) was utilized. Thermal evaporator TE-400 was employed to deposit the Au electrode contact. Thereafter, Lake Shore-Model TTPX Cryogenic 6-arm Probe Station with vibration isolation was engaged to obtain the electrical conductivity and metal–oxide–semiconductor field-effect transistor (MOSFET) data. Finally, Cryogenic Probe Station L419 (LTHMMOS) was used in temperature dependent conductivity.

### Synthesis of ND-Cysteamine

**Figure 1:**
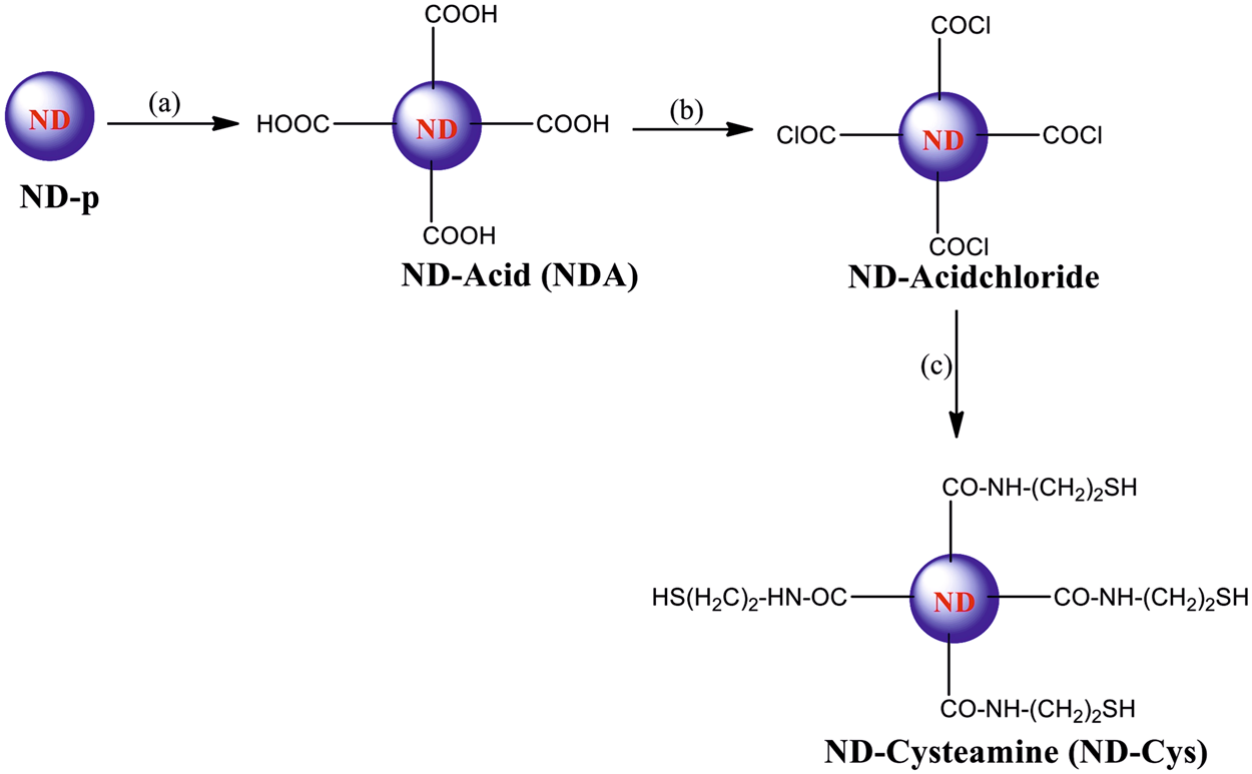
Synthesis of ND-Cysteamine (**a**) H_2_SO_4_:HNO_3_ (9:1), reflux for 12 h; (**b**) SOCl_2_:DMF (10:0.2), reflux for 12 h; (**c**) Cysteamine at pH 10, Toluene, 80 °C for 12 h, stir at pH 3 for 20 min.

As shown in Fig. 1, to 100 mg of commercially purchased nanodiamond powder (ND-p), 40 ml of H_2_SO_4_:HNO_3_ (9:1) was added and refluxed for 12 hours. Then filtered, washed several times with distilled water and dried under vacuum to afford ND-Acid (NDA). Afterwards, the dried NDA was refluxed for 12 hours with 100 mL of SOCl_2_ and 2 mL DMF under inert atmosphere. The brown liquid was decanted and then dried under N_2_ flow to obtain the ND-Acid chloride. Due to the moisture sensitive nature of ND-Acidchloride, it has been utilized directly without any further purification. To the above ND-Acidchloride dispersion in 100 mL of Toluene, excess amount of Cysteamine dissolved in 15 mL Toluene maintained at pH 10 was added and stirred at 80 °C for 12 hours. In the above step, the pH of dissolved Cysteamine solution was maintained by stirring the mixture for an hour with 0.5 mL of Triethylamine and 0.2 mL of 1 M NaOH in ethanol. Thereafter, **ND-Cys** was obtained by removal of excess Cysteamine through several time washing with ethanol followed by stirring at pH 3 for 20 minutes and dried under vacuum. Overall, these synthesis evidenced the affordable yields in all steps^40^.

### SEM and DLS data

For SEM, EDX, Zeta potential and DLS studies on ND-p NDA and **ND-Cys** in 10 microgram (µg) of those derivatives were dispersed in 1 mL of water and utilized as such. Conversely, SEM and EDX of **ND-Cys** nanowires (DNWs) were exploited by 100 nano gram (ng)/mL (Fig. S10, Supporting Information) and 10 picogram (pg)/mL dispersion (Figs S12 and S13, Supporting Information) in pH buffers, respectively. During SEM and EDX measurements, the samples were drop casted on Si-wafer, dried at 50 °C for 15 minutes before data collection.

### TEM and AFM studies

For TEM and AFM analysis of ND derivatives (ND-p, NDA and **ND-Cys**), 10 pg/mL dispersion was considered. Similarly, for HR-TEM invetigation of **ND-Cys** agglomeration, 500 µg in 1 mL water was used. On the other hand, TEM images of G-DNWs were taken by dispersing 1 femtogram (fg) of **ND-Cys** in 1 mL of pH 6 buffer. For AFM, the dispersed **ND-Cys** was drop casted over Si-wafer substrate. Whereas, TEM investigations were performed on the dispersion of ND samples in Copper-Carbon grid.

### FTIR and Raman spectra

For FTIR and Raman analysis, the samples (10 µg/mL) were drop-casted on Si-wafers, then annealed at 60 °C for 30 minutes and continue towards analysis. We used clean Silicon wafer as background reference for both spectroscopy measurements.

### pH induced synthesis of G-DNWs

For this study, 3–11 pH buffers were freshly prepared each time as per the literature^41, 42^ and to cross check our results, comercially available pH buffers were purchased from Merck. Initially, **ND-Cys** NPs (10 µg) were dispersed in 1 mL of 3–11 pH buffers and after uniform incubation for 24 hours diluted the dispesion concentration to 100 ng/mL in respective pH buffers. Then directly proceeded to SEM analysis to evaluate the reproducibility from 20 collected data. The % reproducibility was calculated as follows. Under similar pH buffers 20 vials with ND-Cys dispersion were incubated for 24 hours. Then all samples at similar dispersive conditions were drop casted on the well cleaned Silicon wafers, which were further subjected to Microscopic investigations. Under the Microscope, those wafers were investigated for G-DNWs formation at five different locations in each wafer and the results were accounted to calculate the reproducible nanowires formation. At pH 6, reproducibility seems to be higher due to the effective formation of sp^2^ graphite layer over the DNWs along with impurity channels. Hence, the above procedure was continued to generate the less aggregated and scattered G-DNWs formation at pH 6 with dispersion dilution to 10 pg in 1 mL of pH buffer. For TEM studies, from the above uniform homogeneous dispersion, concentration was further decreased to 1 fg/mL in DI-water.

### Stability of G-DNWs

The above wet synthesized G-DNWs are seems to be highly stable in respective pH buffers (Fig. S11) and upon dispersion in more amount of DI-water (Fig. S17; Supporting Information), the longer nanowires (initially at ~100 µm) were break into small wires/rods (few microns) as illustrated. If, further dispersed and incubated in DI-water for longer time, it will easily break into aggregated particles. However, upon placing the grown G-DNWs into the solution of different pHs, those G-DNWs are seems to be stable between pHs 5 ~ 8 and got affected to break into smaller nanorods or agglomerated nanoparticles in pHs 3, 4, 9, 10 and 11. Notably, the fabricated G-DNW was seems to be highly stable while ultrasonication with water and acetone.

### XRD, Raman and FTIR Investigations on G-DNWs

The XRD of ND-p, NDA and **ND-Cys** were directly taken through powder XRD analysis. On the other hand, the G-DNWs powder were generated by stirring the **ND-Cys** powder in pH 6 buffer for 24 hours. Then, centrifuged and washed several times with DI-water to remove the pH buffer completely, dried in oven under vaccum. The dried G-DNWs powder subjected to XRD analysis. Above G-DNWs powder dispersed in water and drop casted on cleaned Si-Wafer and then subjected to Raman and FTIR investigation as well.

### Fabrication of a single G-DNW

The fabrication of a single G-DNW was done by selecting a well grown nanowire at 10 pg of **ND-Cys** incubated homogeneously in 1 mL of pH 6 buffer for about 72 hours. Which was further diluted to 100 fg/mL in DI water as shown in Fig. S17 (Supporing Information). Furthermore, the cleaning process in each step of single G-DNW fabrication was involved the ultrasonication with Acetone, DI water and then followed by N_2_ purging to rid of water. For single G-DNW device fabrication, the steps follwed are (1) Selection of a high doped N-type silicon wafer as a substrate; (2) Deposition of SiO_2_ (300 nm) on the Si-substrate by PECVD (Temperature:300 °C, RF plasma: 25 W, Chamber pressure: 1000mTorr, SiH4:9 sccm, N2O:710 sccm, reaction time:250 seconds); (3) Completion of outside pad (150 µm*150 µm; Fig. S16a, Supporting Information) *via* cleaning process, spin coating the photoresist, exposure to mask alignment system (DUV lithography) for 10 sec and finally lift off; (4) Completion of alignment mark (Fig. 16b, Supporting Information) through cleaning process, spin coating the photoresist, E-beam lithography and then lift off; (5) Deposition of dispersed G-DNWs at 100 fg/mL of water (Fig. S17, Supporting Information); (6) SEM observation of the nanowire distribution; (7) Utilization of Focus Ion Beam to fix the single G-DNW as well as contact improvement; (8) Completion of electrode connection by means of cleaning process, spin coating the photoresist, E-beam lithography followed by thermal evaporation of Ti/Au (20/100 nm) and finally lift off. The above fabricated device was used in current-volatage (I–V) and temperature dependent conctuctivity as well as MOSFET measurements.

### Activation Energy (E_a_) calculation

The activation energy of diamond nanowire portion L2 was calculated by

1 $${\bf{R}}{\boldsymbol{=}}{{\bf{R}}}_{{\bf{0}}}{\bf{e}}{\bf{x}}{{\bf{p}}}^{{\boldsymbol{(}}{{\bf{E}}}_{{\bf{a}}}{\boldsymbol{/}}{\bf{k}}{\bf{T}}{\boldsymbol{)}}}$$

Where R and R_0_ are the resistance of L2 at applied and zero voltage, k is known as Boltzmann’s constant and T is temperature in kelvin (k). Upon ploting “lnR Vs 1000/T” (Fig. S18, Supporting Information), the activation energy (E_a_) can be obtained from the slope^43^.

## Results and Discussions

As shown in Fig. 1, the brief synthetic procedure^40^ was followed to obtain **ND-Cys**. In which, upon treating the ND-pristine (ND-p) with H_2_SO_4_:HNO_3_ (9:1), ND-Acid (NDA) has been obtained. Afterward, in presence of catalytic amount of dimethyl formamide (DMF) NDA reacted with thionyl chloride (SOCl_2_) to afford the moisture sensitive ND-Acidchloride. Those humidity sensitive ND-Acidchloride derivative was directly used without any further purification. It was treated with excess amount of cysteamine (maintained at pH 10), followed by stirring at pH 3 for 20 minutes to provide the compound **ND-Cys** with an affordable yield. In order to avoid the competing reaction with thiol (-SH) group, the cysteamine has been maintained at pH 10 for an hour, which may led to its dimeric form cystamine. On the other hand, stirring of final product at pH 3 buffer for 20 minutes could led to regeneration of free thiol group again^44, 45^.

Then, **ND-Cys** was characterized by Fourier Transform Infrared (FTIR) and Raman spectral investigations. As noticed in Fig. S1 (supporting information), the –C = O stretching band of **ND-Cys** was displayed at 1600 cm^−1^ contrast to ND-p (1639 cm^−1^) and NDA (1610 cm^−1^). The –OH of ND-p and –COOH of NDA were broadened at 3433 and 3393 cm^−1^, respectively. Whereas for **ND-Cys**, the amide –NH broad band was displayed at 3414 cm^−1^. Further, the –CH stretching of ND-p and **ND-Cys** were correspondingly appeared at 2928 and 2950 cm^−1^. Impressively, presence of free thiol (–SH) group in **ND-Cys** was approved by the band at 2578 cm^−1^ along with amide –C-N stretching band at 1510 cm^−1^. Similar to FTIR, Raman spectra also confirm **ND-Cys** formation. As seen in Fig. S2 (Supporting Information), ‘D’ and ‘G’ band of ND-p, NDA and **ND-Cys** were evidenced at 1325 and 1600 cm^−1^ along with the partial graphitization of **ND-Cys**. The –CH band of **ND-Cys** was witnessed at 2963 cm^−1^. Additionally, amide –NH bands were broadened at 3210 cm^−1^. Likewise, zeta potential changes (Figs S3–S5, Supporting Information) were confirmed the cysteamine functionalization on nanodiamond surface. Compared to ND-p (−25.29 mV) and NDA (−27.94 mV), **ND-Cys** have positive zeta potential (+10.24 mV), hence supported the functionalization.

**Figure 2:**
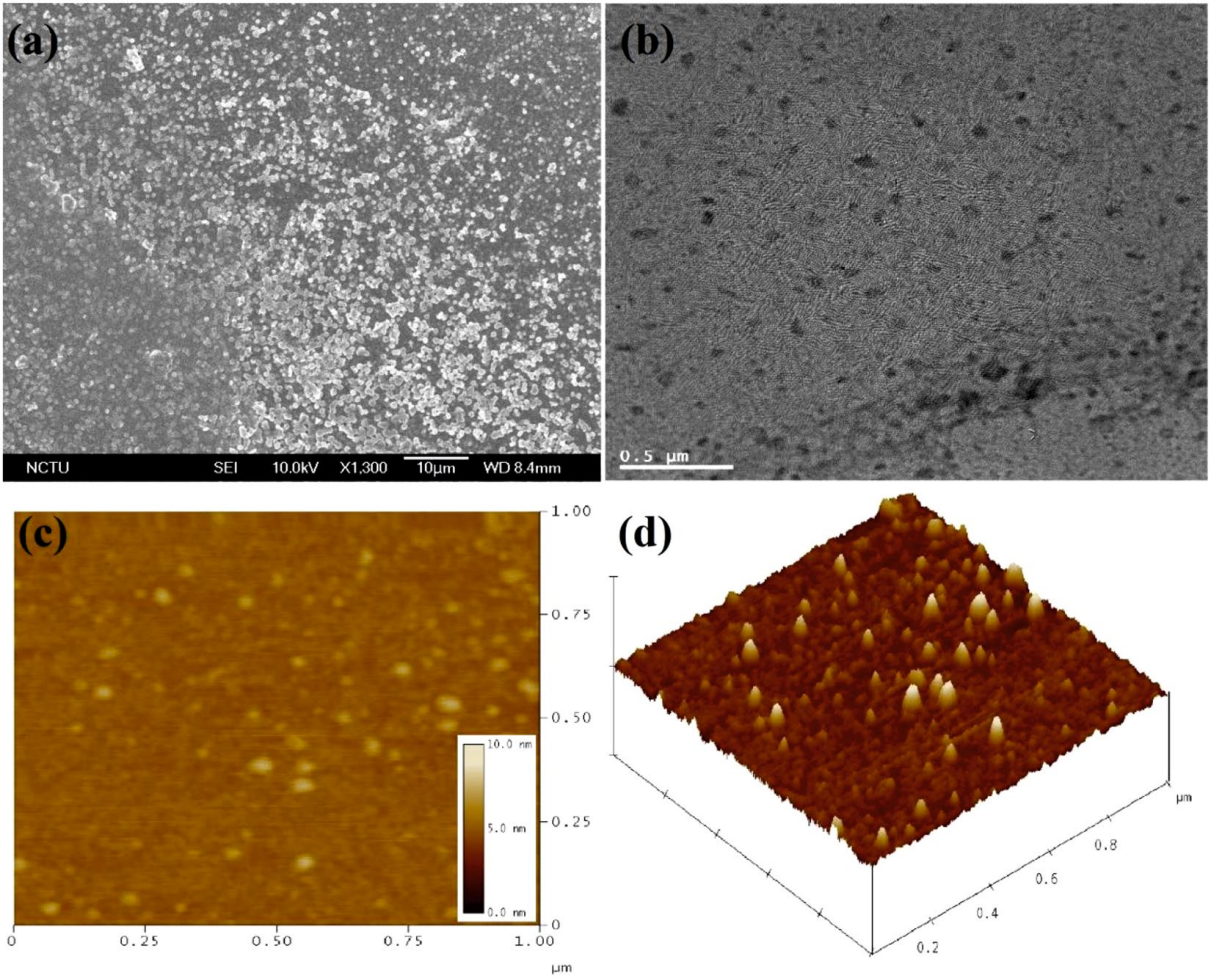
(**a**) SEM image of **ND-Cys** at 10 µg/mL dispersion; (**b**) TEM image of **ND-Cys** at 10 pg/mL dispersion; (**c**) AFM images of **ND-Cys** at 10 µg/mL dispersion and (**d**) AFM height image of **ND-Cys** at 10 µg/mL dispersion.

As shown in Fig. 2a and S6 (Supporting Information), at 10 microgram (µg)/mL dispersion in water, the Scanning Electron Microscopy (SEM) visualize the differential morphology of **ND-Cys** than that of ND-P and NDA. Moreover, the dynamic light scattering (DLS) of NDA and **ND-Cys** (10 µg/mL in water (Fig. S7a and b, Supporting Information), resulted as 85.6 ± 50.7 and 159.6 ± 94.3 nm, respectively, hence also held the cysteamine functionalization. Thereafter, the elemental concentration was estimated by energy dispersive spectroscopy (EDX), as presented in Fig. S8a–c (Supporting Information) and Table 1. The presence of S (10.90%) and N (15.85%) atoms with O (16.90%) and reduced content of C (56.35%) were authenticated the cysteamine capping. But, upon varying the dispersive concentration from 10 µg to 10 picogram (pg), the particle size of **ND-Cys** was unable to obtain from DLS data^46, 47^, dispersive concentration, at which G-DNWs were formed. Henceforth, to obtain exact particle size at higher dispersion, Transmission Electron Microscopy (TEM) and Atomic Force Microscopy (AFM) studies were taken into account.

**Table 1:** EDX data of nanodiamond derivatives and G-DNWs.

Compound	C (%)	O (%)	N (%)	S (%)
ND-p	95.35	4.65	—	—
NDA	86.80	13.20	—	—
**ND-Cys**	56.35	16.90	15.85	10.90
**ND-Cys** NWs	47.43	16.37	19.39	16.81

**Figure 3:**
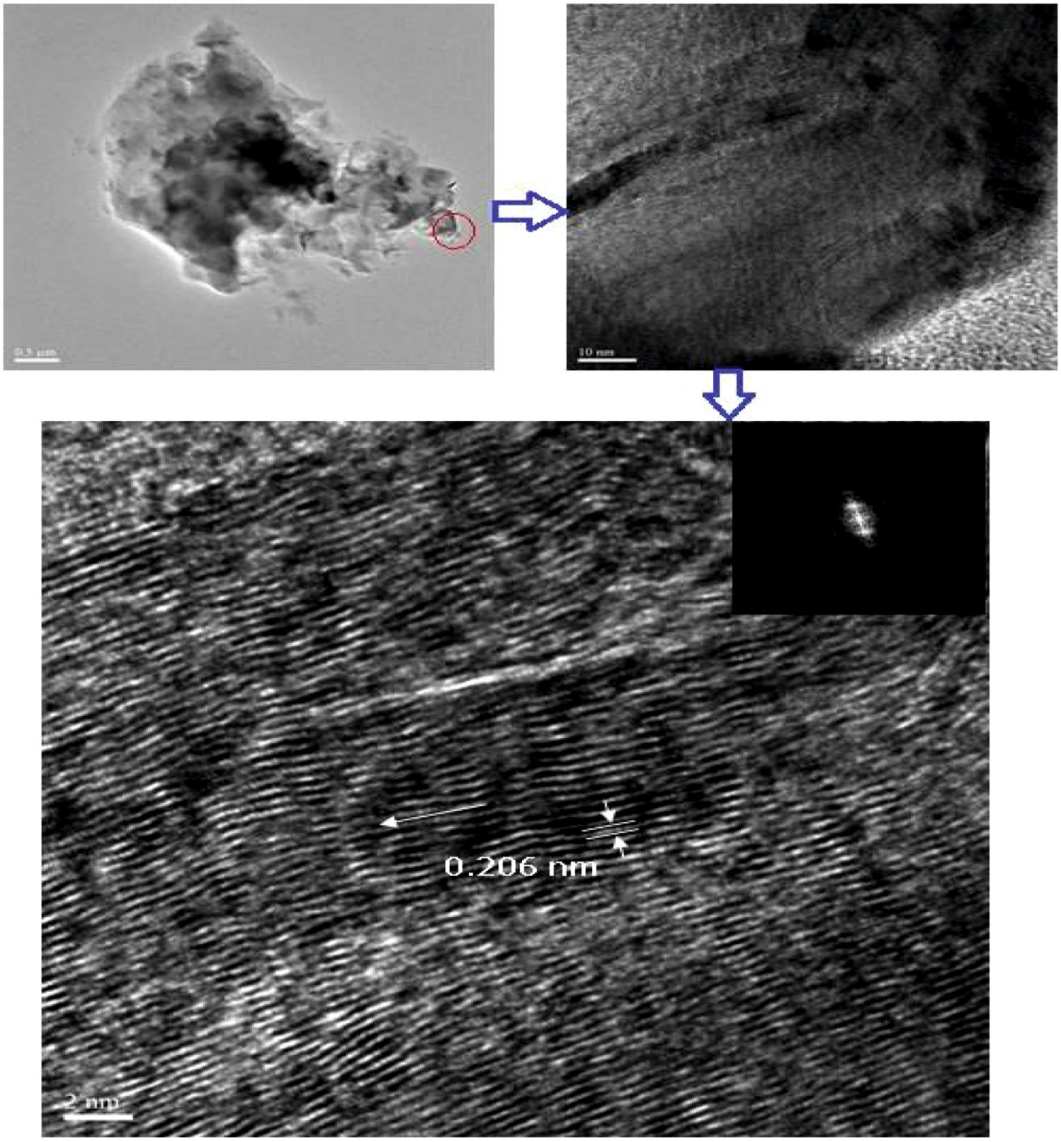
HRTEM of **ND-Cys** agglomeration represents partial graphitization and the diffraction distance 0.206 nm related to (111) pattern of nanodiamond.

TEM images of ND-p, NDA and **ND-Cys** at 10 pg/mL dispersion were envisioned in Fig. 2b and S9 (Supporting Information). From which, the particle sizes of NDA and **ND-Cys** seems to be smaller than that of DLS value. In case of **ND-Cys** (10 pg/mL; Fig. S9e and f, Supporting Information), the particle size might be stuck between 20 ~ 250 nm. Above result was validated by AFM image (Fig. 2c), which evident the possible small particles of **ND-Cys**. Likewise, the AFM height image (Fig. 2d) has also been established the possible small particles along with large sized particles. Moreover, as evident by Raman studies, the high resolution-TEM (HR-TEM) image of **ND-Cys** at high concentrated agglomerate state was also validated the partial graphitization as exposed in Fig. 3. Further to note, the diffraction distance 0.206 nm was corresponds to nanodiamond (111) pattern^48^. In addition, the TEM also tells about the existence of defects or impurity channels in the grains of diamond nanoparticles. Those defects or impurities may arose from the wet chemical synthesis of DNPs and are responsible for the formation of less perfect sp^2^ graphite layer over DNWs as discussed in lateral sections.

**Figure 4:**
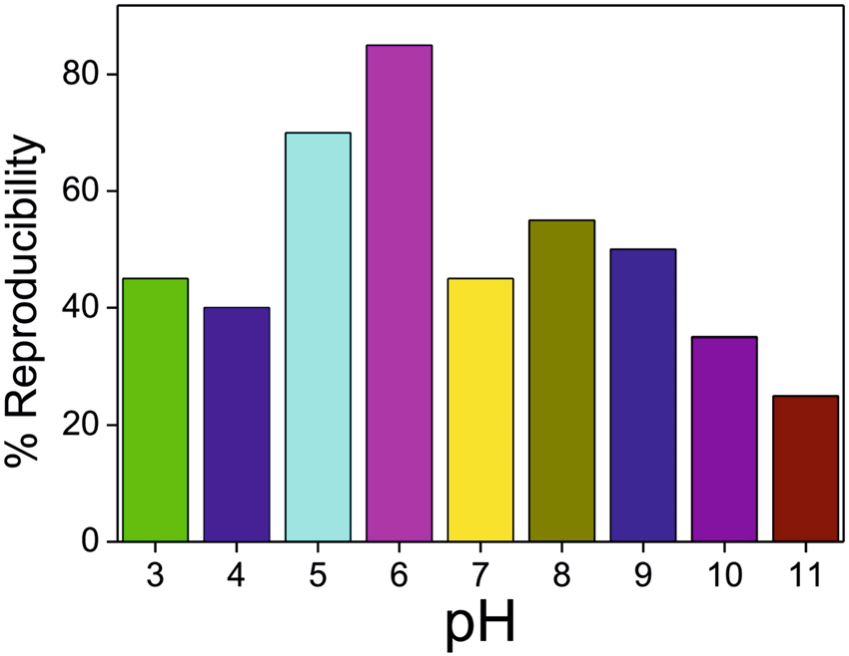
Reproducibility percentage from 20 collected data on DNWs growth with respect to pHs.

Afterwards, focused on the development of graphitized hybrid G-DNWs *via* tuning pHs from 3 to 11 by maintaining 100 nanogram (ng)/mL dispersion as demonstrated by SEM (Fig. S10, Supporting Information). But, after 24 hours, about 85% reproducibility (from 20 collected data) on G-DNWs formation were witnessed at pH 6 contrast to other pHs as exposed in Fig. 4. Here, apart from those reproducible data, 15% of partial G-DNWs formation were notified, which required the higher time incubation for its completion. On the other hand, prolonged dispersion of **ND-Cys** (up to 1 week) in pH 6 may enhanced the reproducibility up to 95%. Therefore, extended G-DNWs construction only at pH 6 by further diminishing the dispersion concentration as depicted in Fig. S11 (Supporting Information).

**Figure 5:**
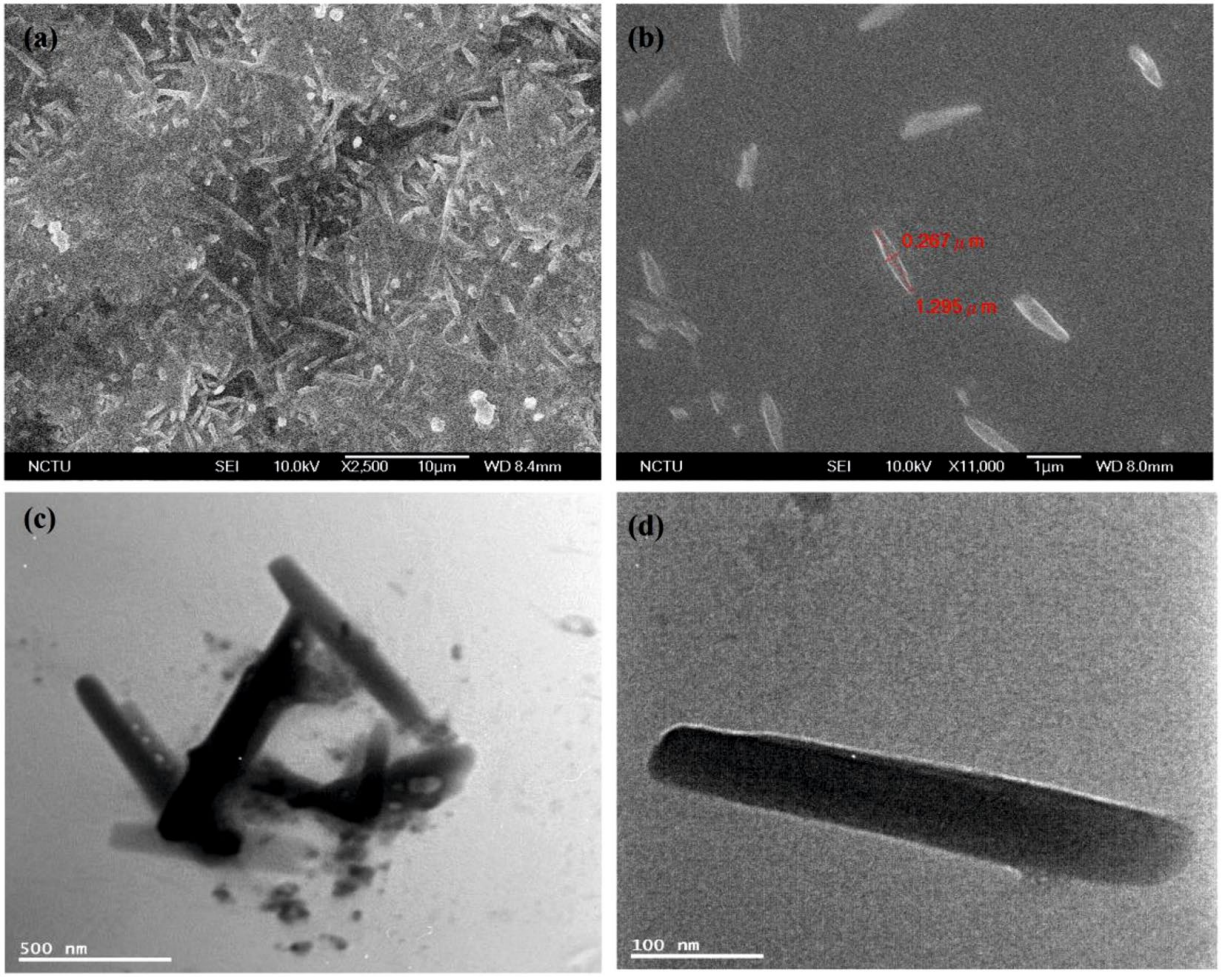
(**a**,**b**) SEM images of **ND-Cys** NWs (10 pg/mL in water) at different regions and (**c**,**d**) TEM images of **ND-Cys** nanowires (1 fg/mL in water) at different regions.

At pH 6, the width of G-DNWs ranges between 20 to 800 nm and length lies between 200 nm ~ hundreds of microns reliant on dispersion concentration. Contrary to so far used diamond nanowires of 5–20 nm diameter, due to the poly-disperse nature of **ND-Cys** NPs, we obtained the G-DNWs with higher diameter. These poly-disperse nature was attributed to the intramolecular H-bonds present between small nanowires to assemble to become a DNW with higher diameter^49^. While varying the dispersion concentration of **ND-Cys** at this pH, the scattered G-DNWs with limited lengths were envisaged by SEM [10 pg/mL; Fig. 5a,b and S12a–c (Supporting Information)] and TEM [1 femtogram (fg)/mL; Fig. 5c,d and S12d–f (Supporting Information)].

In a similar way, G-DNWs with small diameter can be obtained by its dispersion from femtogram (10^15^ g) in pH buffer to attogram (10^18^ g) in water. Incredibly, the EDX spectrum of DNWs at pH 6 (Fig. S13, Supporting Information) evident the improved percentile of S (19.39%) and N (16.81%) atoms along with reduced content of O (16.37%) and C (47.43%) than that of origin as presented in Table 1. Hence, confirmed the involvement of S and N elements in G-DNWs formation. Further to note, as mentioned earlier, maybe those impurity channels or defects may formed through dislocation of S and N atoms of Cysteamine from core to the surface of G-DNWs (at pH 6) via electrostatic self-assembly of ND-Cys with the support of H-bonding as recognized in EDX pattern. Which might led to the development of less perfect sp^2^ graphite layer over DNWs. Additionally, the –SH and –NH involved in intramolecular H-bonds with –C=O may enhance the conductivity on the surface as in the case of proteins and peptides^50^.

The mechanism behind these G-DNWs formation might be as follows. At pH 6, the DNWs formation was initiated through electrostatic forces within the partially graphitized ND-Cys particles, which may further stabilized through the intramolecular H-Bonds (on the surface of diamond Nano particles) between the amide –C=O with either –NH or –SH groups. During the above interactions, pH 6 was employed as initiative source to enhance DNWs formation and it also boost the formation of graphene shells with impurity channels. Moreover, the partially graphitized ND-Cys particles and defects/impurity channels (obtained from synthesis) were further promoted to form the graphene shells on the surface of DNPs and sandwiched between the diamond cores. Which was tend to generate the less perfect sp^2^ graphite layer over DNWs. Presence of those graphene shells on the less perfect graphite layer and the impurity channels were act as the connecting units between diamond cores of the DNWs. Notably, due to the electrostatic self-assembly of different sized diamond nanoparticles, the thickness of graphite sheath/graphene shells may vary throughout the DNWs. Hence, those graphene shells and impurity channels were plays the vital role in the diverse electrical transport as explained latter.

**Figure 6:**
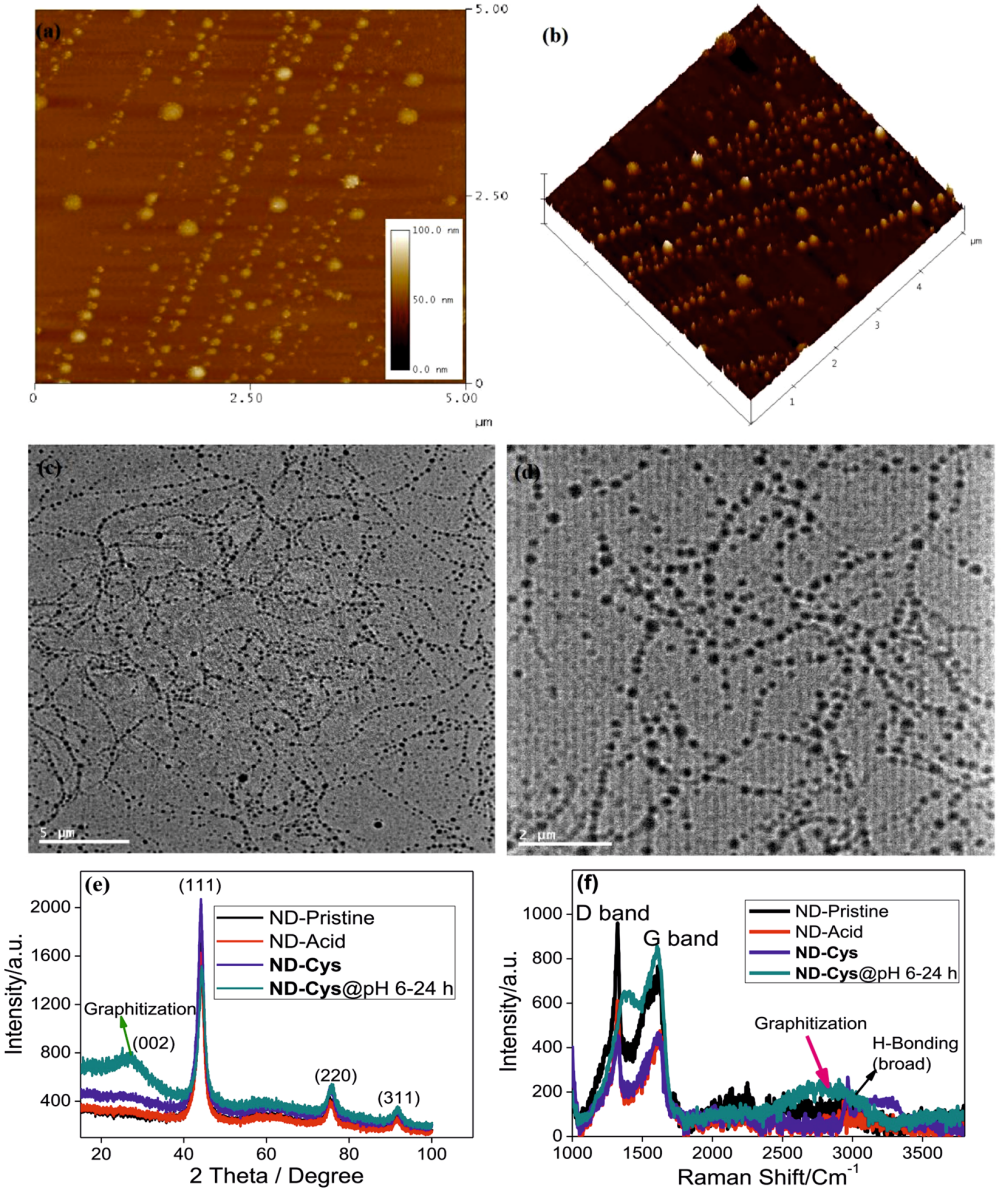
(**a**) AFM image of **ND-Cys** self-assembly after 3 hours at pH 6; (**b**) AFM height image representation of self-assembly; (**c**,**d**) TEM images representing the self-assembly of **ND-Cys** after 3 hours at pH 6; Scale bars: 5 µm and 2 µm, respectively; (**e**,**f**) XRD and Raman spectra on **ND-Cys** graphitization.

**Figure 7:**
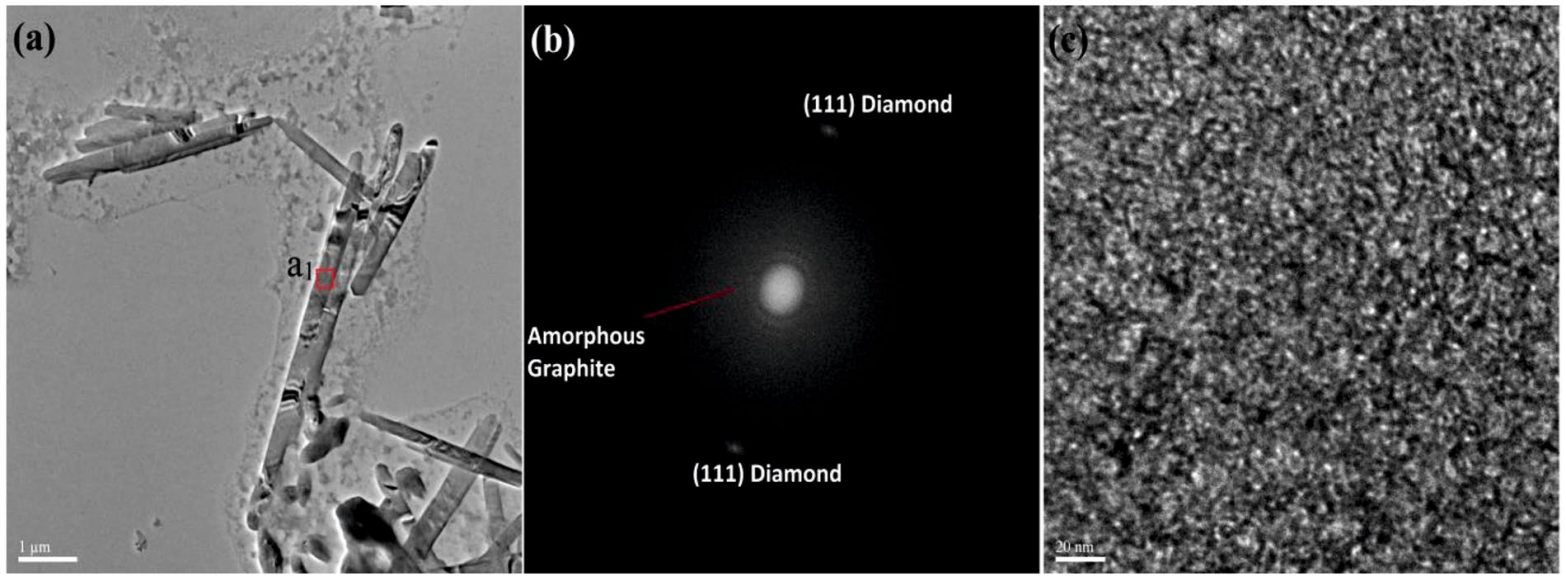
(**a**) HR-TEM image of G-DNWs (**b**) FT pattern of selected area **a**_**1**_ representing amorphous graphite along with diamond (111) diffraction pattern and (**c**) High magnification image of **a**_**1**_ region representing less perfect graphite layer along with defects or impurity channels.

To verify the self-assembly process, AFM and TEM studies were undertaken. Surprisingly, those investigations were revealed the self-assembly of **ND-Cys** at pH 6 even after 3 hours. As noticed in Fig. 6a and b, the AFM images were well evidenced the self-assembly of **ND-Cys** after 3 hour incubation at pH 6. Which was further extended to form the G-DNWs after 24 hours. In a similar manner, the TEM studies also well confirmed the self-assembly of **ND-Cys** as exposed in Fig. 7c and d. Then, graphitization over DNWs was established by powder X-ray diffraction analysis (XRD), Raman and FTIR investigations. As illustrated in Fig. 6e, the XRD pattern of diamond derivatives were observed at 2θ = 44.01, 75.61 and 91.34 related to (111), (220) and (311) planes. However, upon incubating **ND-Cys** at pH 6 for 24 hours notified the newer 2θ peak at 26.60, resemble formation of graphite (002) layer^51^ over diamond core. Similarly, Raman spectra also evidenced the upshifted high intense ‘G’ band at 1596 cm^−1^ and downshifted low intense ‘D’ band at 1363 cm^−1^ than that of **ND-Cys**, as shown in Fig. 6f. Moreover, due to the graphitization and H-bonding, a broad band between 2400 ~ 3200 cm^−1^ was witnessed. Akin to above studies, FTIR (Fig. S14, Supporting Information) also verified the graphitization and H-bonding by diverse broad bands at 1900 ~ 2500 and 2600 ~ 3700 cm^−1^. Furthermore, the upshifted –C=O and –NH stretching peaks at 1571 and 1412 cm^−1^ were supported H-bonds involvement in graphitization.

Next, to demonstrate the graphitization over DNW, HR-TEM investigation were carried out. As noticed in Fig. 7a and b, the HR-TEM and FT diffraction analysis of a G-DNW illustrate the existence of amorphous graphite sheath and (111) diamond diffraction pattern as well as the wrapping of more amorphous graphite sheath above the diamond core. In which, upon higher magnification visualization in TEM (Fig. 7c), the diamond diffraction pattern was not been observed. This might be due the coexistence of diamond core along with amorphous graphite grains and impurity channels generated in synthesis as well as in pH buffer induced G-DNWs growth. On the other hand, the HR-TEM images exposed in Fig. S15a and b (Supporting Information), also establish the differential graphitization on DNWs grown by these wet chemical route. Wherein, only amorphous graphite was detected in that FT diffraction studies. Similarly, upon tuning to high magnification, the impurity channels or defective voids were also envisaged as displayed in Fig. S15c (Supporting Information). It suggest that, contrary to CVD based G-DNWs, above DNWs have less perfect sp^2^ graphite layer over diamond core and each wires may have diverse graphite sheath wrapping. Hence, possibly portions of DNWs must the dissimilar conductivity in I-V measurements as discussed next. Ultimately, many attempts to visualize the graphite sheath and diamond core of G-DNWs via HR-TEM studies were repeatedly affected by the presence of amorphous less perfect sp^2^ graphitic layer and impurity channels over sp^3^ diamond carbon. Therefore, we stretched our focus to conductivity studies as noted below.

**Figure 8:**
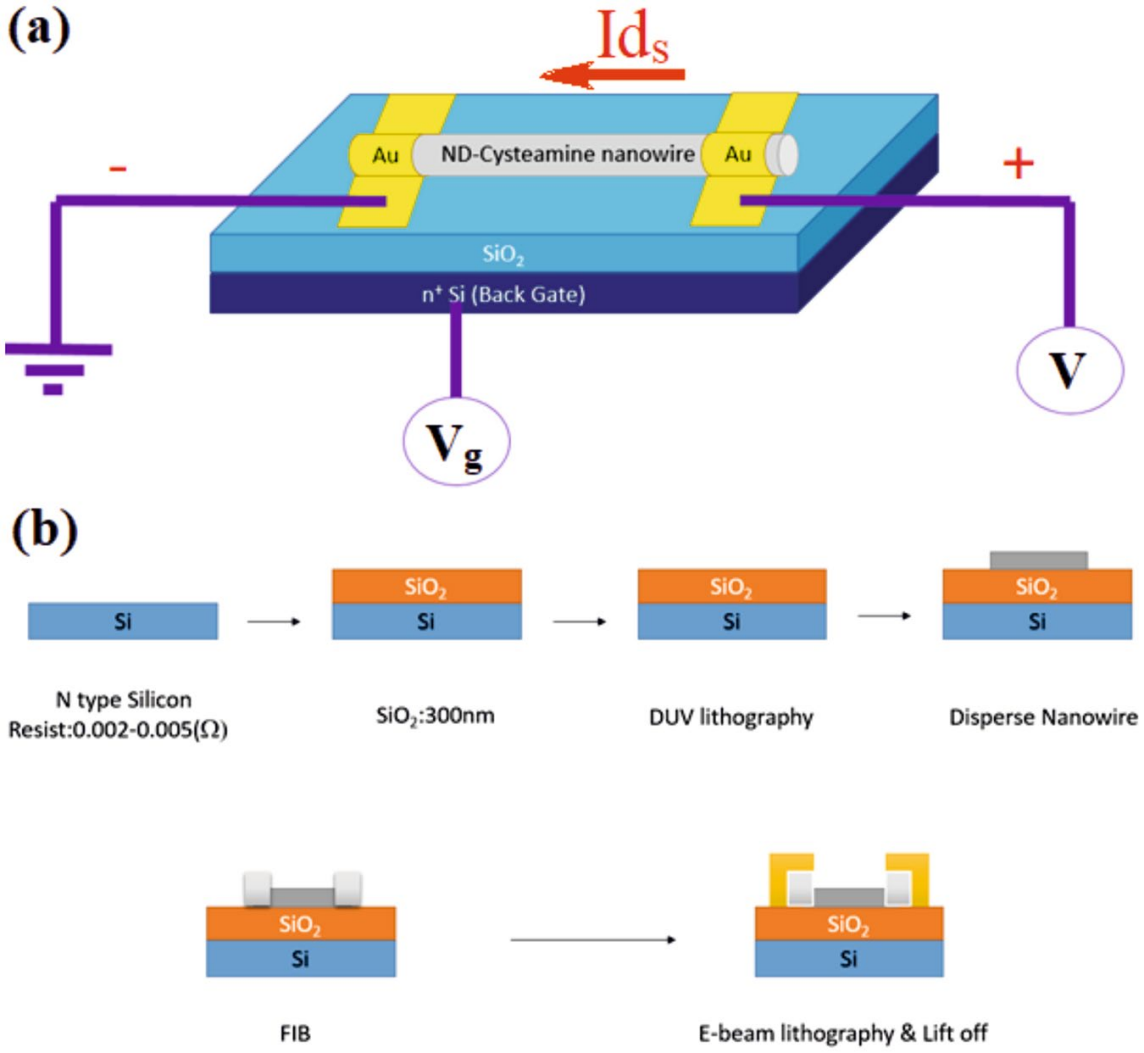
(**a**) Device Schematic of a single G-DNW fabrication and (**b**) Fabrication steps involved in single **ND-Cys** NW device.

**Figure 9:**
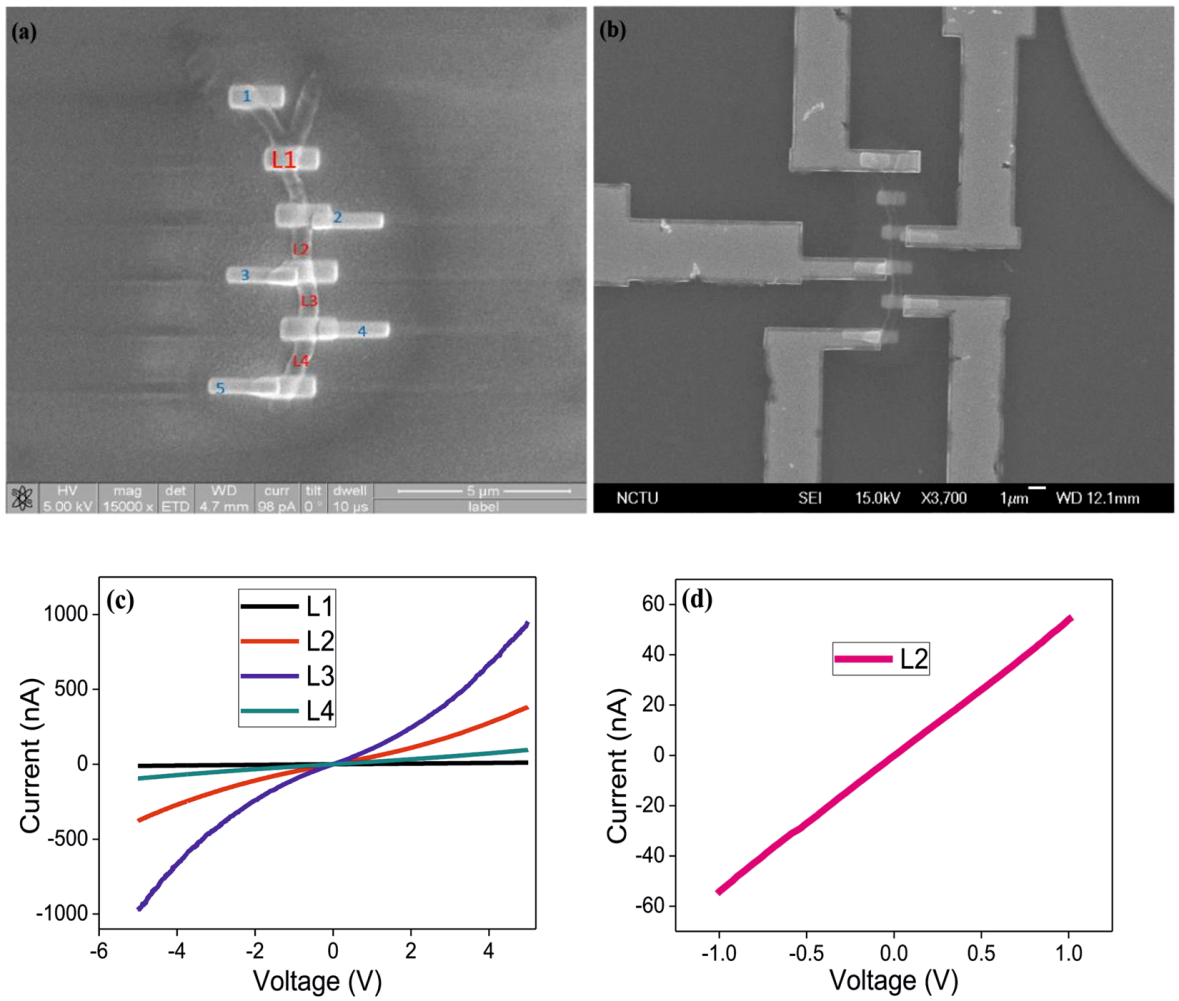
(**a**,**b**) SEM images of single **ND-Cys** NW with Au contacts; (**c**) Two-point probe current-voltage (I–V) curves of L1, L2, L3 and L4 and (**d**) Four-point probe current-voltage (I–V) curve of L2.

To well verify the hybrid G-DNWs formation, conductivity studies on a chosen single DNW has been undertaken by following the fabrication process and device schematic as illustrated in Fig. 8a and b. Above fabrication process involved the pad and alignment mark followed by DNWs dispersion (Figs S16 and S17, Supporting Information). The device structure is demonstrated as N-Si/SiO_2_/**ND-Cys** NW/Au and the selected single G-DNW with Au electrode contact is shown in Fig. 9a. As depicted in Fig. 9b, the G-DNW length portions between the contacts 1 to 5 has been classified as L1(3.8 µm), L2(1 µm), L3(1 µm) and L4(1.2 µm). The selected G-DNW have the width of 500 nm and radius (r) of 250 nm.

The conductivity of L1, L2, L3 and L4 were established from two and four point probe I–V measurements and the calculations^52^ were carried out by

2 $${\boldsymbol{\sigma }}{\boldsymbol{=}}1{\boldsymbol{/}}{\boldsymbol{\rho }}$$

where σ denotes the conductivity in S/cm and ρ is known as static resistivity obtained from

3 $${\boldsymbol{\rho }}{\boldsymbol{=}}{\bf{R}}({\bf{A}}{\boldsymbol{/}}{\bf{l}})$$

Here, ‘R’ represents electrical resistance of a specimen in Ω, ‘A’ is the cross-sectional area of the specimen in m^2^ and ‘l’ is the length of material in meter.

**Figure 10:**
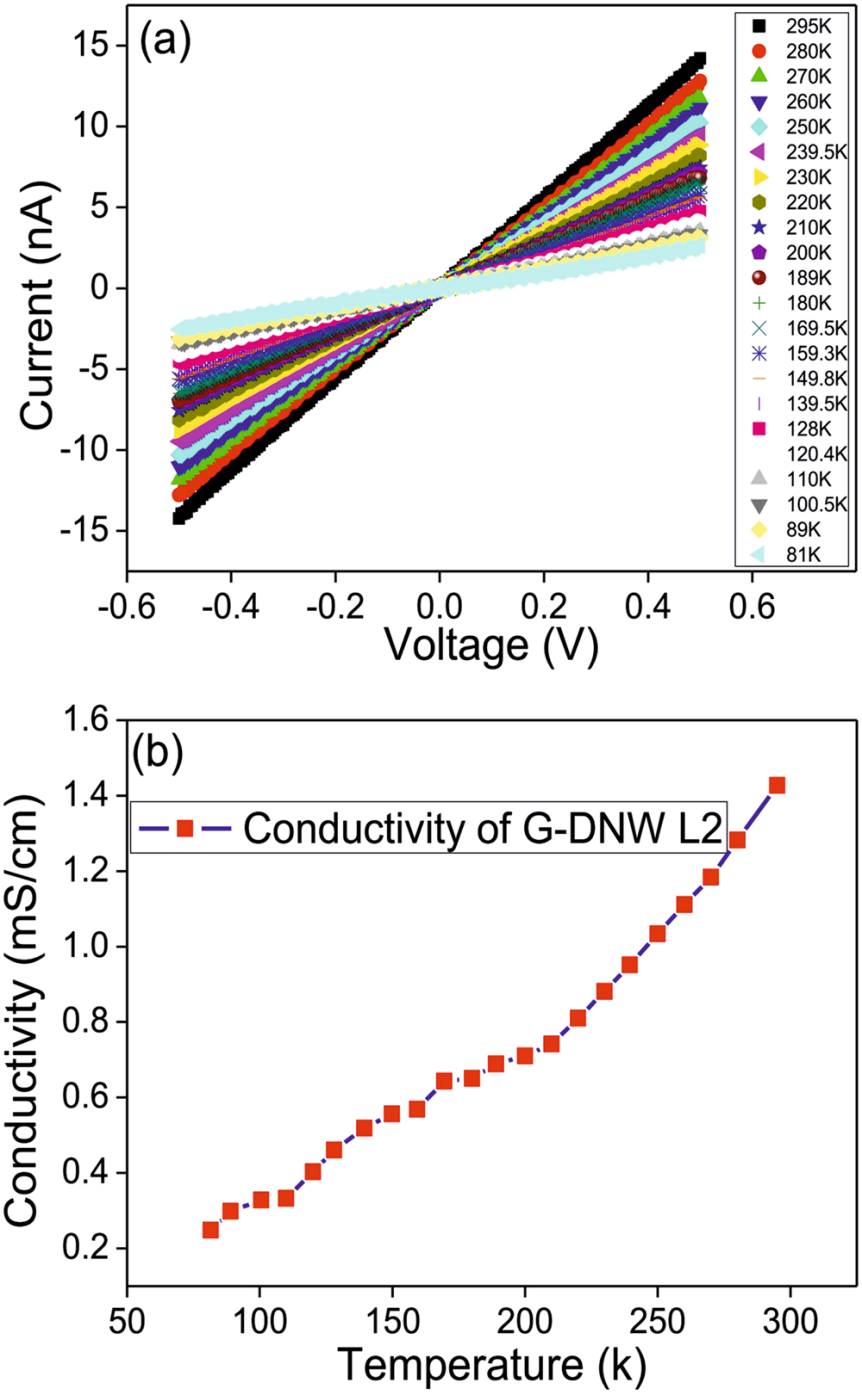
(**a**) Temperature dependent current-voltage (I–V) curves for L2 between 80 ~ 295 k and (**b**) Temperature dependent conductivity of L2.

As shown in Table 2, the calculated conductivity (between −5 to 5 V) from Fig. 9c, notified that L2 and L3 have the better conductivity of 2.4 ± 1.92 × 10^−6^ and 4.8 ± 1.83 × 10^−5^ mS/cm than that of L1 and L4. Because of irregular graphite sheath growth on L1 and L4 portions, the conductivity of them are become different. This might be due to the electrostatic self-assembly of different sized diamond nanoparticles to provide the diverse thickness of graphite sheath/graphene shells coverage over L1 and L4. Above statement was also authenticated by the HR-TEM studies on G-DNWs. Additionally, four point probe I–V measurement (Fig. 9d) was calculated the intrinsic resistivity of L2 as 370.755 ± 0.437 Ω-cm. To interrogate the temperature dependent conductivity, I–V measurements between 81~295 K were engaged, which evidenced the down fall in conductivity with respect to decrease in temperature as seen in Fig. 10a and b.

**Table 2:** Resistance, resistivity and conductivity data of L1, L2 L3 and L4 area of a selected **ND-Cys** nanowire.

DNW Area	Resistance (MΩ)	Resistivity (Ω-cm)	Conductivity (mS/cm)
L1	487(0.01% ± 0.0487)	2516.37(0.01% ± 0.25)	0.397(0.01% ± 3.97 × 10^−8^)
L2	21.27(0.08% ± 0.02)	417.64(0.08% ± 0.33)	2.4(0.08% ± 1.92 × 10^−6^)
L3	10.61(0.38% ± 0.040)	208.32(0.38% ± 0.79)	4.8(0.38% ± 1.83 × 10^−5^)
L4	63(0.03% ± 0.0189)	1030.83(0.03% ± 0.31)	0.97(0.03% ± 2.91 × 10^−7^)

In addition, I–V characteristics also showed linear and symmetrical behavior through the entire temperature range, which indicated that the contacts are ohmic and these conductivity was originated from the sp^2^ graphitic carbon present on the G-DNW surface. Furthermore, it may be attributed to the sulfur (S) and Nitrogen (N) atoms of cysteamine present on the surface of G-DNWs. Likewise, a plot of lnR *vs* 1000/T (Fig. S18, Supporting Information) elucidate the activation energy (E_a_) of L2 as 40 MeV^43^. Impressively, electron transport mechanism has been interpreted from plots of “lnR *Vs* 1/T” and “conductance *Vs* T^−(1/4)^” *via* nonlinear and linear fittings, respectively, as shown in Fig. S19 (Supporting Information) and verified that between 220 ~ 80 K the conductivity was due to mott variable-range hopping^53^ and above 220 K, it may arose from thermal activation mechanism^54^. In other words, it has been stated as at room temperature, possibly the electrons hopping were carried through the coexisted impurity channels and graphene shells. Whereas, at high temperature, the electrical conductivity was supported by the thermally activated less perfect sp^2^ graphite layer present over the DNWs. Moreover, attempts on device based metal–oxide–semiconductor field-effect transistor (MOSFET)^55^ discovered that because of low carrier concentration and unsuitable dielectric layer the conductivity of DNW cannot be modulated by the applied gate bias (Fig. S20, Supporting Information). This is partially due to poor carrier concentration in the material. Which can be further modulated by using the appropriate dielectric material in the three-terminal device towards future applications. Further to justify the importance of our conductivity measurement, a comparative account on electrical resistivities of carbon based materials are shown in Table 3.

**Table 3:** Comparative account on electrical resistivities of **ND-Cys** nanowire with respect to selected diamond materials, carbon nanotube and carbon nanowire from I–V measurements.

Materials	Growth Route/Technique	Electrical Resistivity (Ω-cm)
Undoped-UNCD^56^	NA	10^6^
Single Crystal Diamond^57^	Commercial Source	10^14^
Ultra-thin Nanocrystalline Diamond Films^58^	MPCVD	5 × 10^13^
Nitrogen Incorporated Diamond Films^59^	MPCVD	10^5^
Single Diamond Nanowire^60^	APCVD	NA
Carbon Nanotube^61^	Carbon-arc Method	10^−3^~10^−6^
Carbon Nanowire^62^	NA	0.015
Single **ND-Cys** G-DNW	Wet-Chemical Synthesis	370.76^This Work^

NA = Not Available.

In which, our results are lies between diamond carbon nanotube and diamond based electrical resistivity studies. These improved results on single G-DNW device was attributed to the coexistence of less perfect sp^2^ graphitic layer over diamond core along with impurity channels generated in wet synthesis. Hence, open the new window in nanodiamond based semiconductor research applied in future.

## Conclusions

In summary, for the first time nanodiamond particles (ND-Cys) was utilized in pH induced self-assembly driven hybrid graphite-DNWs construction. Interestingly, at pH 6, G-DNWs were formed with better reproducibility. Those DNWs have the width between 20 to 800 nm and its length lies between 200 nm ~ hundreds of microns. In this direction, the graphite wrapping over DNWs was been interpreted from Raman, XRD and TEM interrogations. Moreover, the pH induced self-assembly in G-DNWs construction was well authenticated by TEM and AFM images. Elaborately, a single G-DNW device based conductivity measurement was done to demonstrate the formed hybrid G-DNWs applicability. The single G-DNW portion L2 revealed the better conductivity of 2.4 ± 1.92 × 10^−6^ mS/cm and its temperature dependent carrier transport mechanism was found to be mott variable-range hopping (between 220~80 K) and thermal activation mechanism (above 220 K) with an activation energy of 40 MeV. Importantly, the mechanistic aspects of G-DNWs formation and electrical transport were discussed in detail. These new strategy of G-DNWs synthesis can open the new window on diamond nanowires based semiconductor research.

## Electronic supplementary material

Supplementary Information
