# Supplementary material for: An Affordable Wet Chemical Route to Grow Conducting Hybrid Graphite-Diamond Nanowires: Demonstration by A Single Nanowire Device
Source: Sci Rep. 2017 Sep 11;7:11243. doi: 10.1038/s41598-017-11741-9 (PMC5593905; doi:10.1038/s41598-017-11741-9)
Supplement: Supplementary file 1 — Supplementary Information [file 41598_2017_11741_MOESM1_ESM.pdf]

Supporting Information

An Affordable Wet Chemical Route to Grow Conducting Hybrid Graphite-Diamond Nanowires: Demonstration by A Single Nanowire Device

Muthaiah Shellaiah,[1] Tin Hao Chen,[1] Turibius Simon,[2] Liang-Chen Li,[3] Kien Wen Sun,*[,1,3,4] and Fu-Hsiang Ko[2]

[1]Department of Applied Chemistry, National Chiao Tung University, Hsinchu 300, Taiwan

E-mail: kwsun@mail.nctu.edu.tw

[2]Department of Materials Science and Engineering, National Chiao Tung University, Hsinchu 300, Taiwan.

[3]Center for Nano Science and Technology, National Chiao Tung University, Hsinchu 300, Taiwan.

[4]Department of Electronics Engineering, National Chiao Tung University, Hsinchu 300, Taiwan.

Table of contents:

FTIR and Raman spectra of ND-p, NDA and **ND-Cys (S3-S4)**

Zeta Potentials of ND-p, NDA and **ND-Cys (S5-S7)**

SEM of (a) ND-p, (b) NDA and (c, d) **ND-Cys** dispersion at 10 µg/mL in water **(S8)**

DLS of (a) NDA and (b) **ND-Cys** dispersion at 10 µg/mL in water **(S9)**

EDX spectra of (a) ND-p, (b) NDA and (c) **ND-Cys (S10)**

TEM of (a, b) ND-p (c, d) NDA (e, f) **ND-Cys** at 10 pg/mL dispersion in water **(S11)**

SEM images of diamond nanowires (G-DNWs) formation at pH buffers 3 to 11 **(S12)**

Dilution representation of **ND-Cys** dispersion at pH 6 **(S12)**

SEM (10 pg/mL) and (d-f) TEM (1 fg/mL) of **ND-Cys** at pH 6 after 24 hours **(S13)**

EDX spectrum of **ND-Cys** nanowires (G-DNWs) **(S13)**

FTIR spectra of **ND-Cys** dispersion in pH 6 after 24 hours **(S14)**

HR-TEM of ND-Cys Nanowires **(S14)**

Images of pad and alignment mark **(S14)**

Representation of **ND-Cys** NW dispersion for device fabrication **(S15)**

Activation energy ($E_{a)}$ calculation from “lnR *Vs* 1000/T” plot **(S15)**

Plots of “Conductance *Vs* 1/T” and “lnR Vs $T^{(-1/4)}$” **(S16)**

MOSFET based conductivity measurements **(S17)**

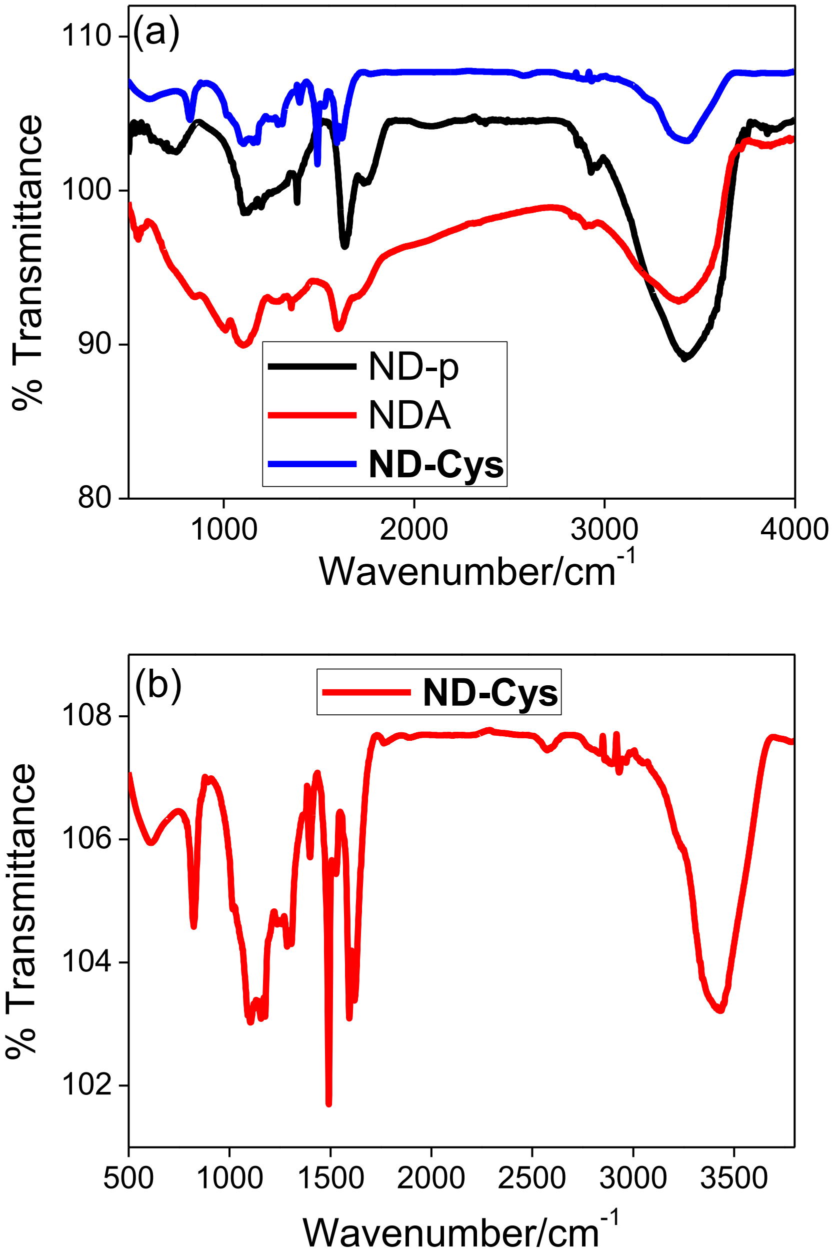

Figure S1. (a) FTIR spectra of ND-p, NDA and **ND-Cys**; (b) FTIR spectrum of **ND-Cys**.

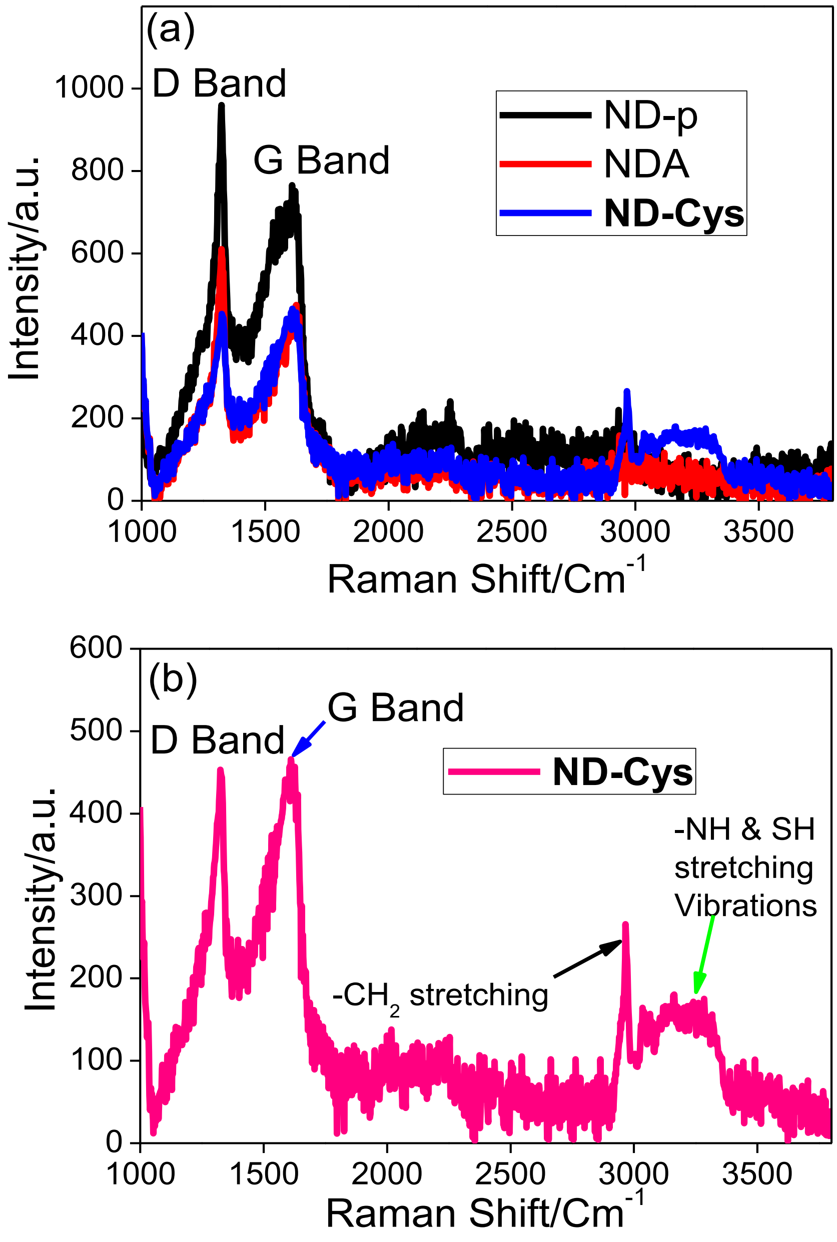

Figure S2. (a) Raman spectra of ND-p, NDA and **ND-Cys**; (b) Raman spectrum of **ND-Cys**.

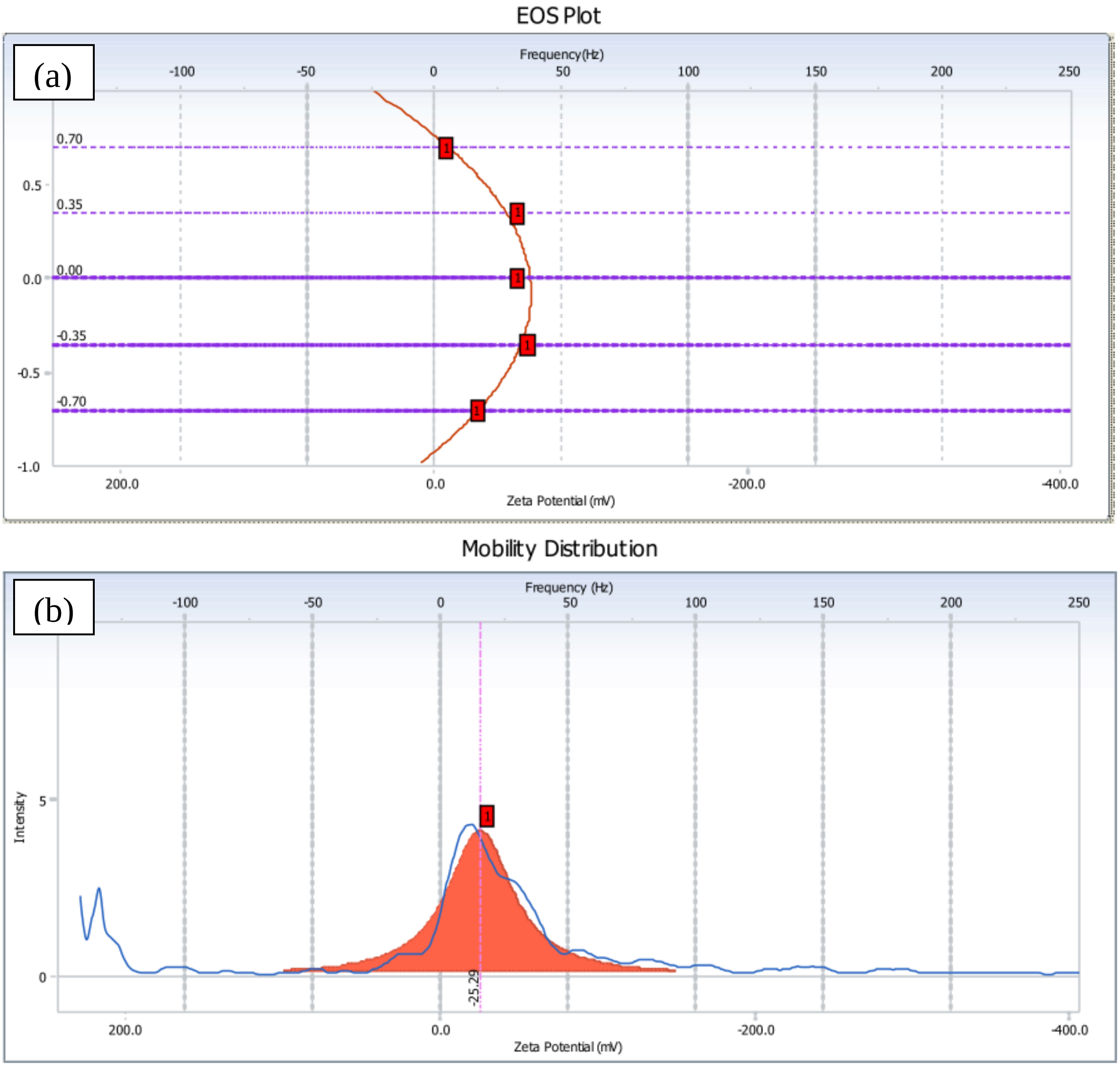

Figure S3. (a, b) Zeta Potential of ND-p dispersion at 10 µg/mL in water.

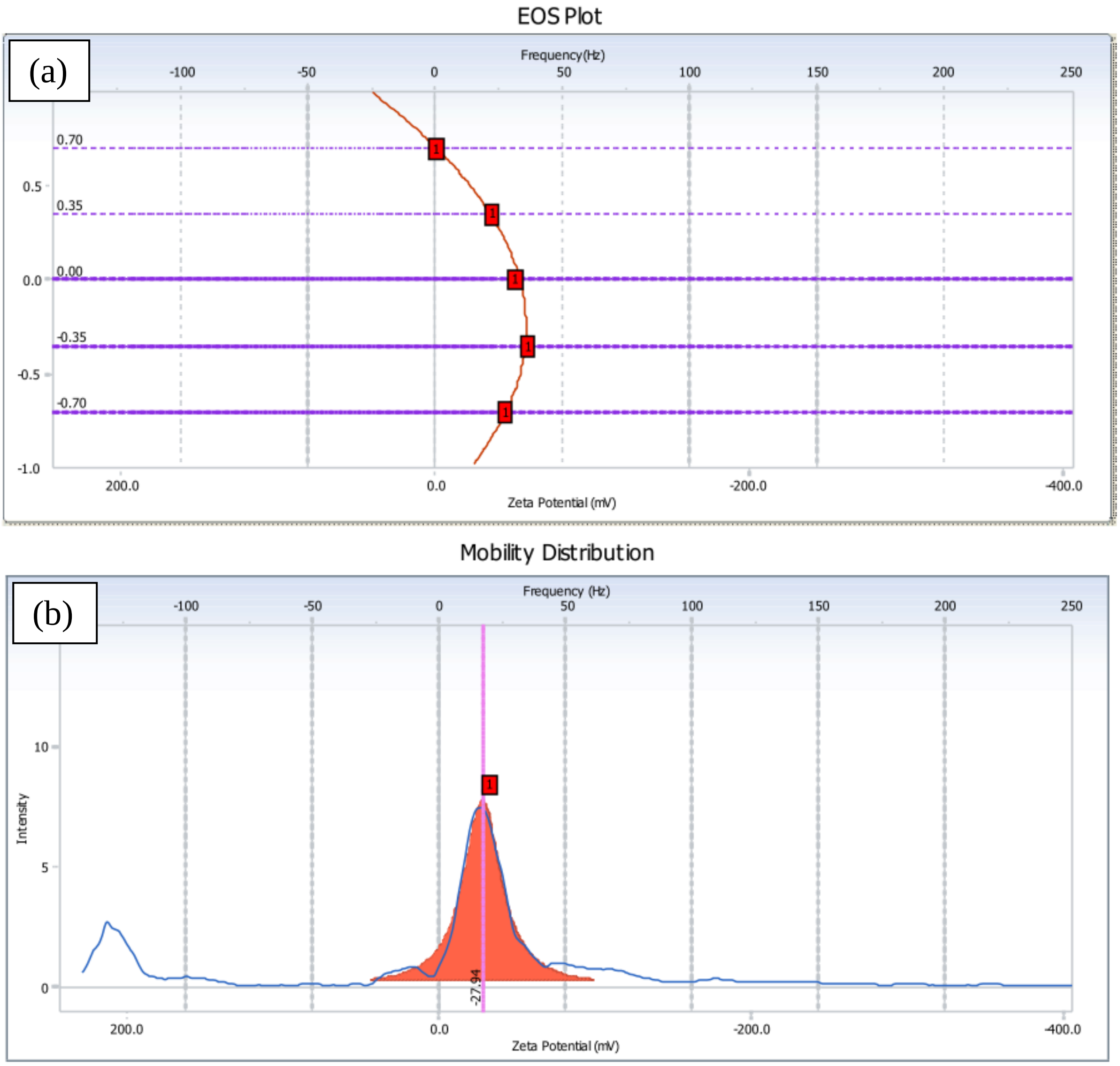

Figure S4. (a, b) Zeta potential of NDA dispersion at 10 μg/mL in water.

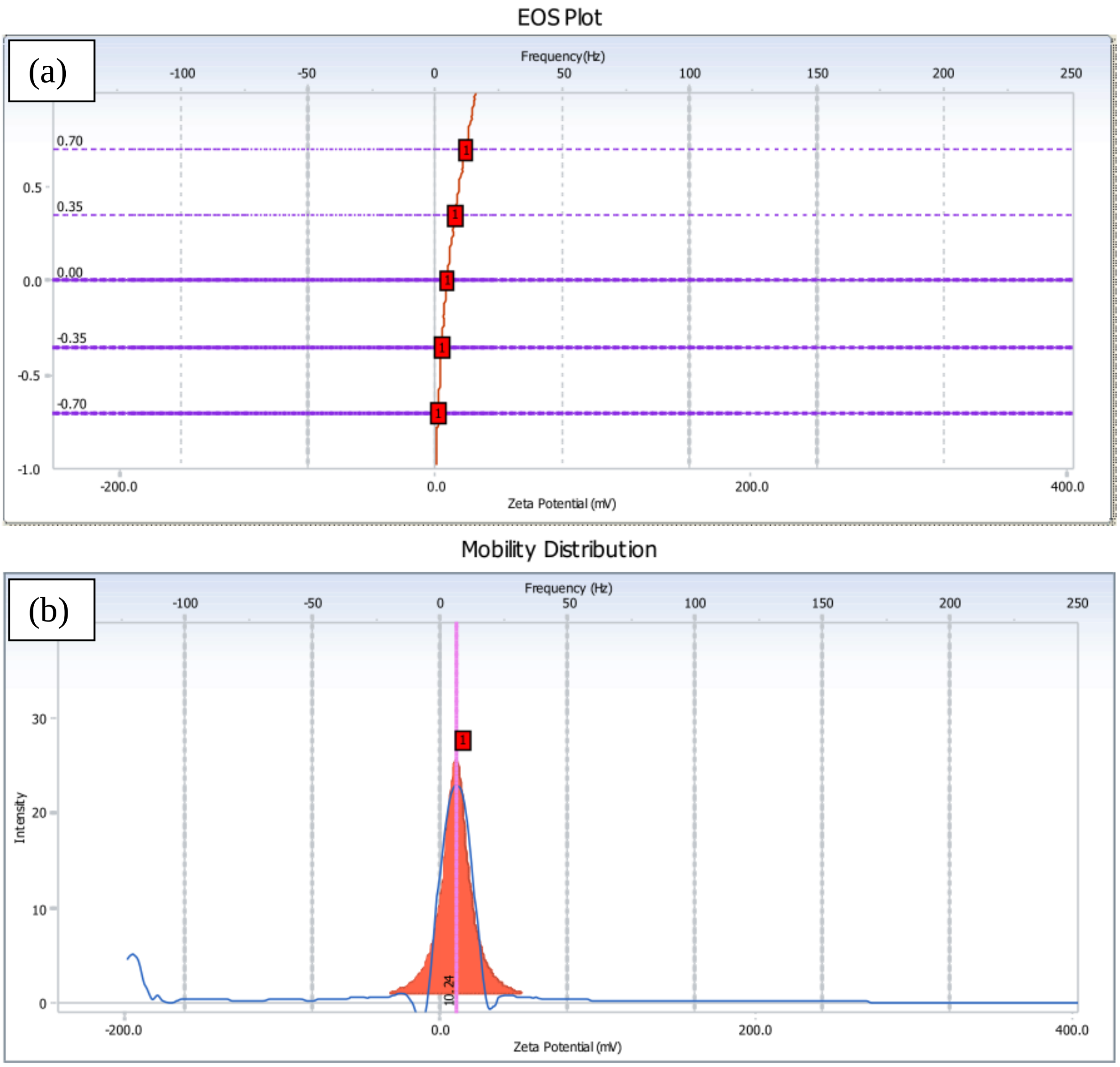

Figure S5. (a, b) Zeta potential of **ND-Cys** dispersion at 10 µg/mL in water.

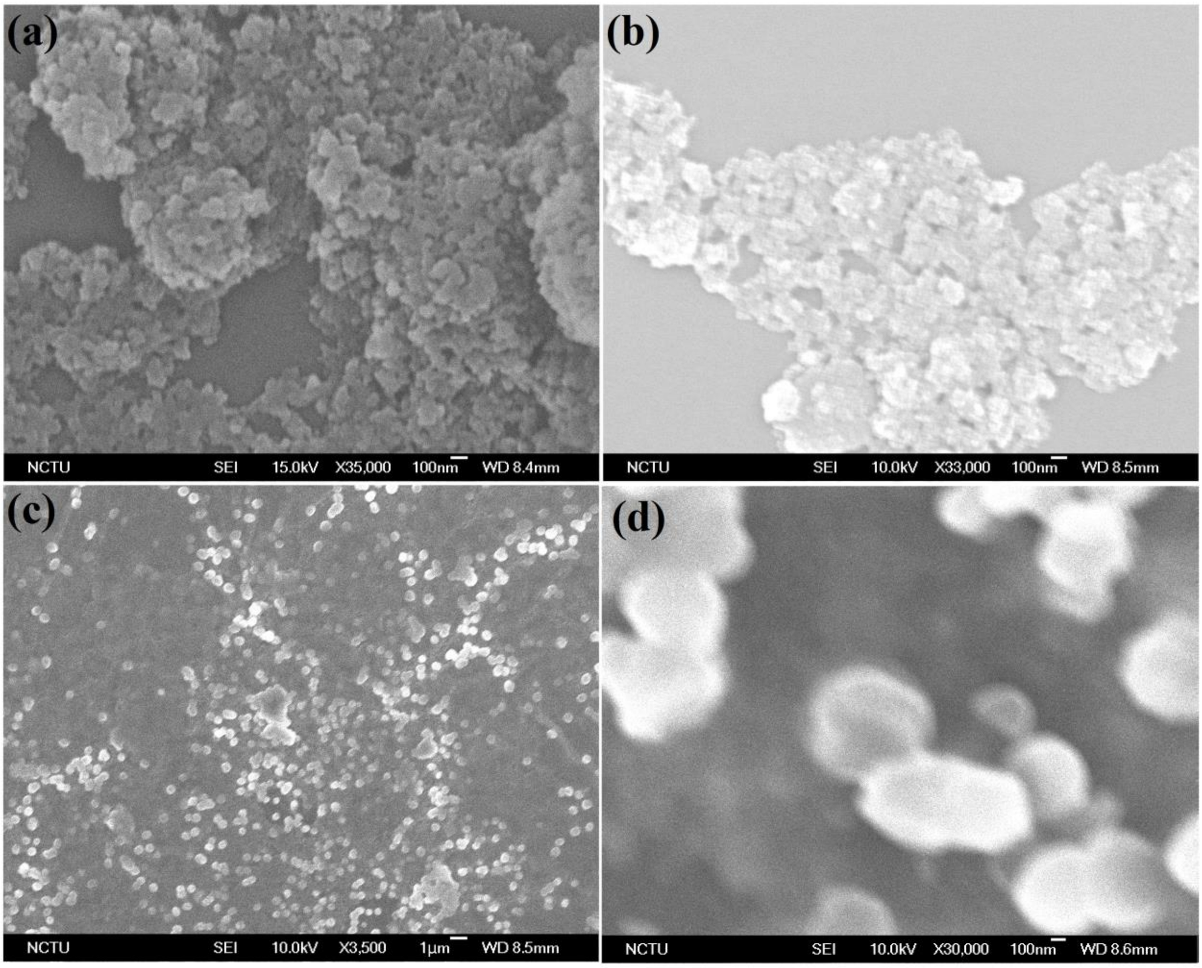

Figure S6. SEM of (a) ND-p, (b) NDA and (c, d) **ND-Cys** dispersion at 10 µg/mL in water.

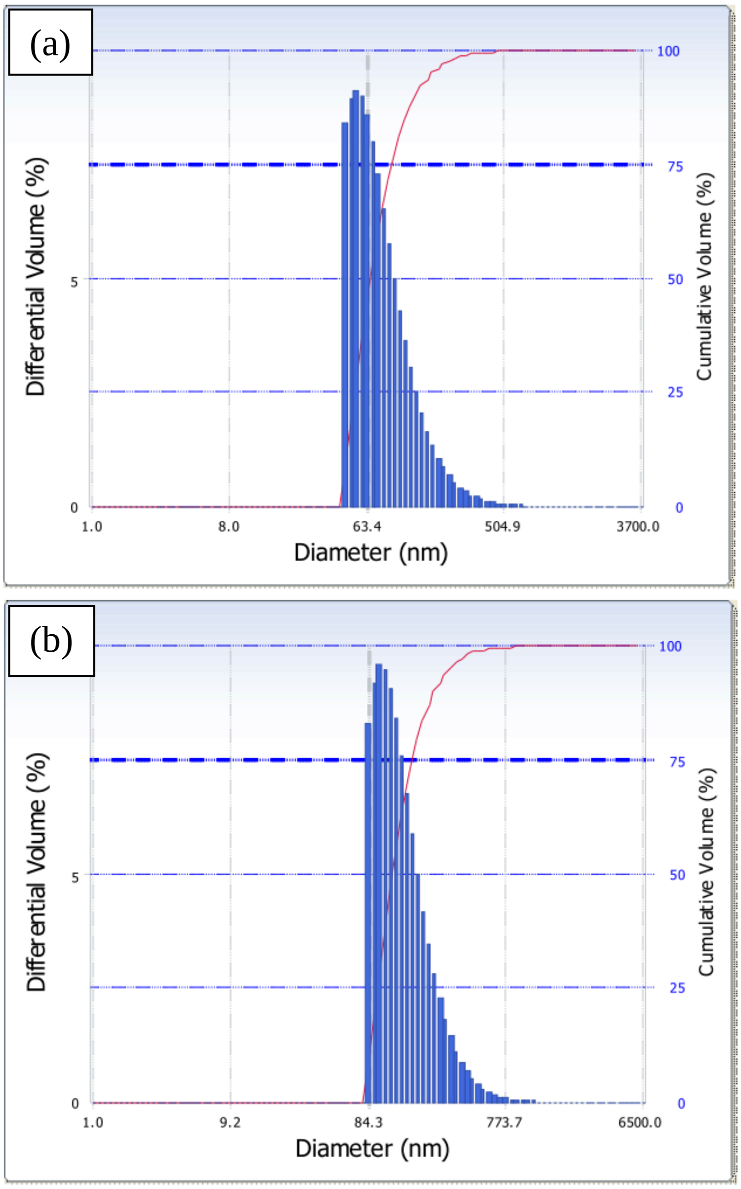

Figure S7. DLS of (a) NDA and (b) **ND-Cys** dispersion at 10 µg/mL in water.

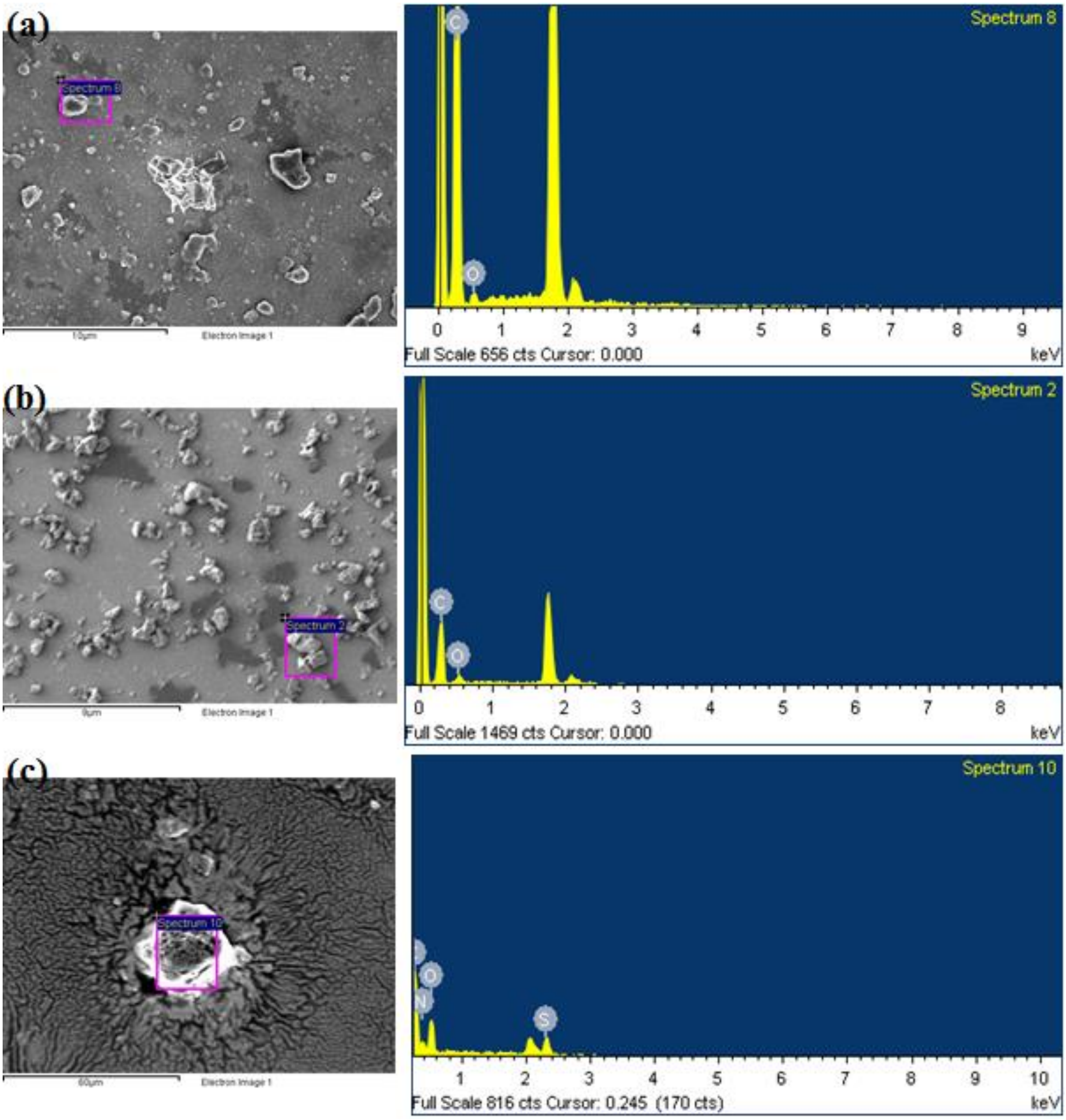

Figure S8. EDX spectra of (a) ND-p, (b) NDA and (c) **ND-Cys**.

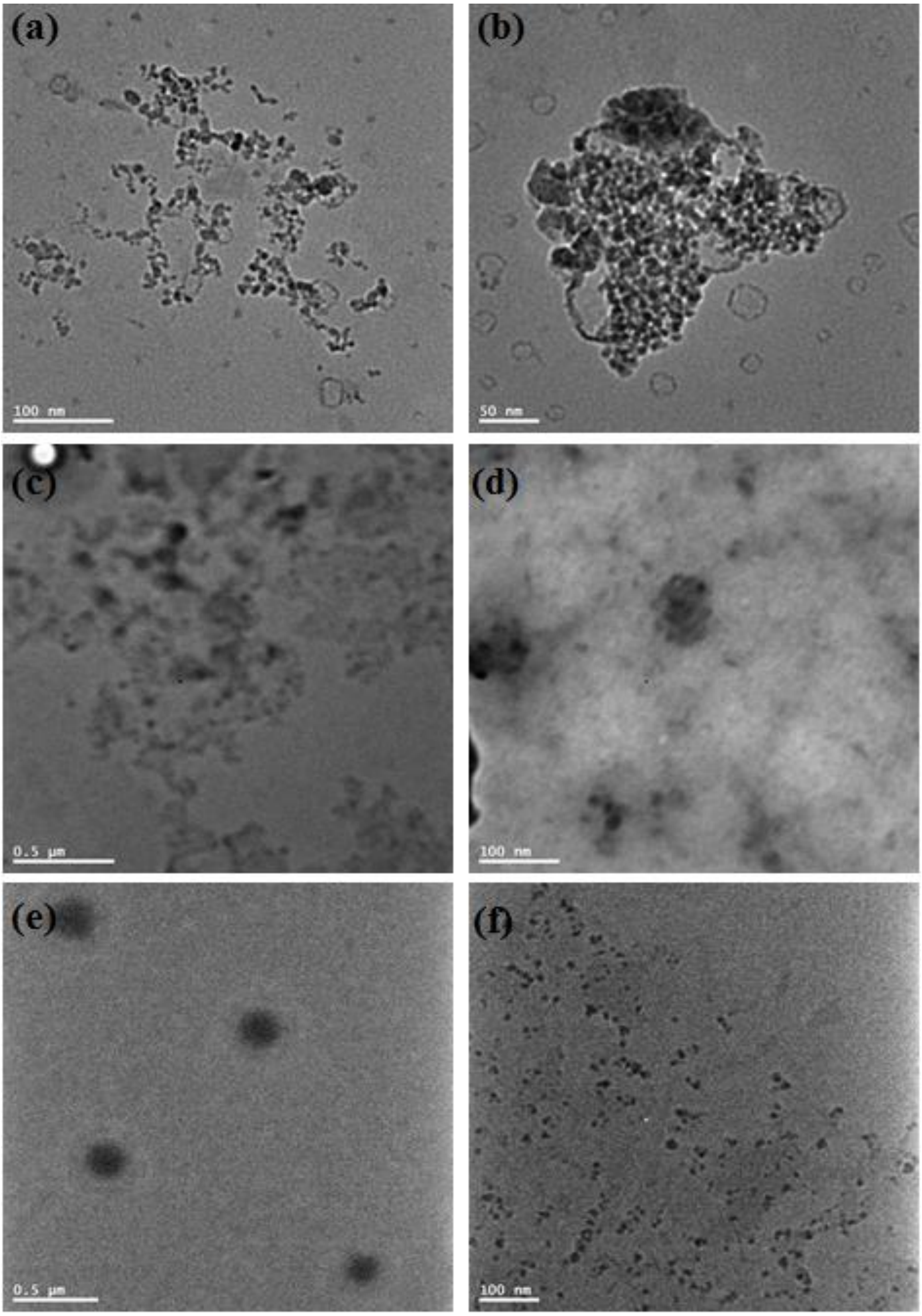

Figure S9. TEM of (a, b) ND-p (10 pg/mL in water), (c, d) NDA (10 pg/mL in water) (e, f) **ND-Cys** at 10 pg/mL dispersion in water, examined at different regions.

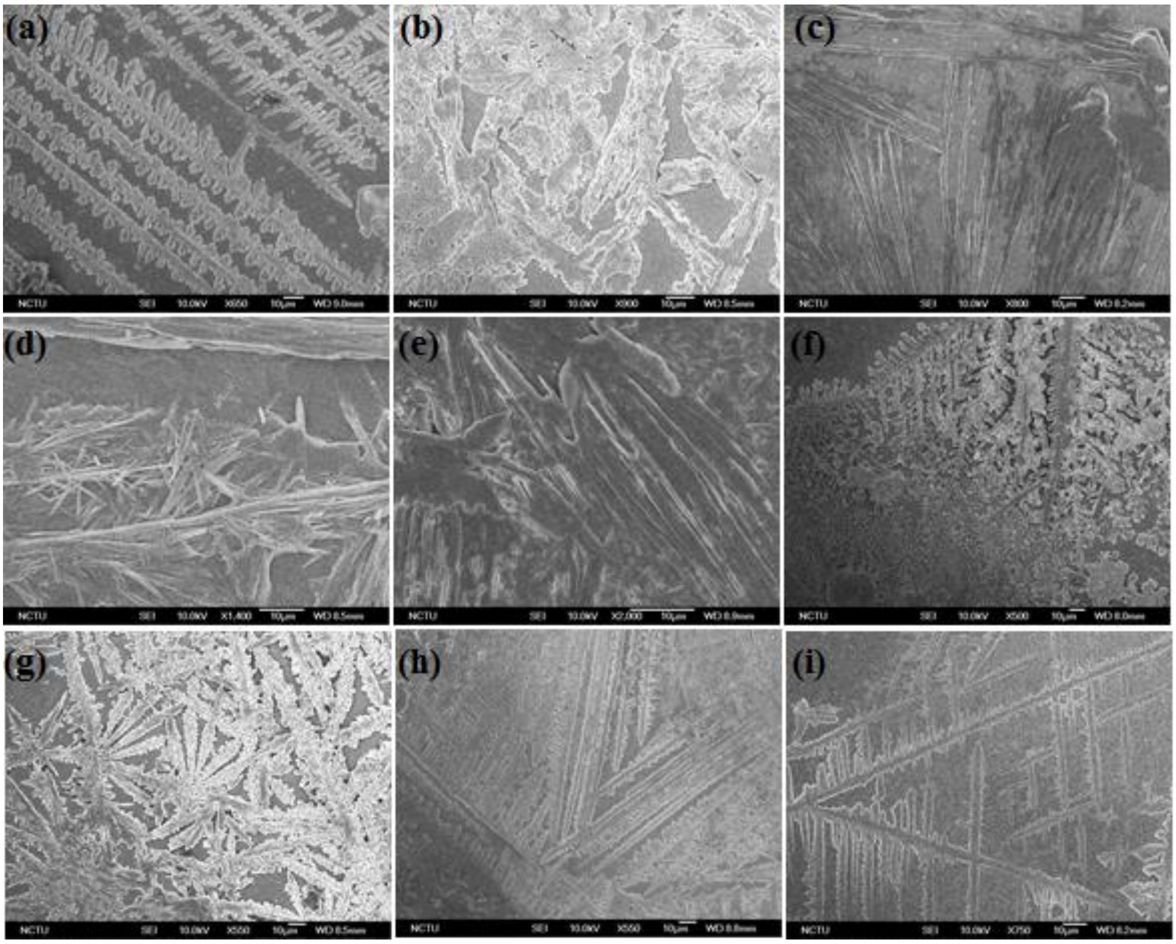

Figure S10. SEM images of diamond nanowires (G-DNWs) formation from **ND-Cys** dispersion at 100 ng/mL in pH buffers 3 to 11.

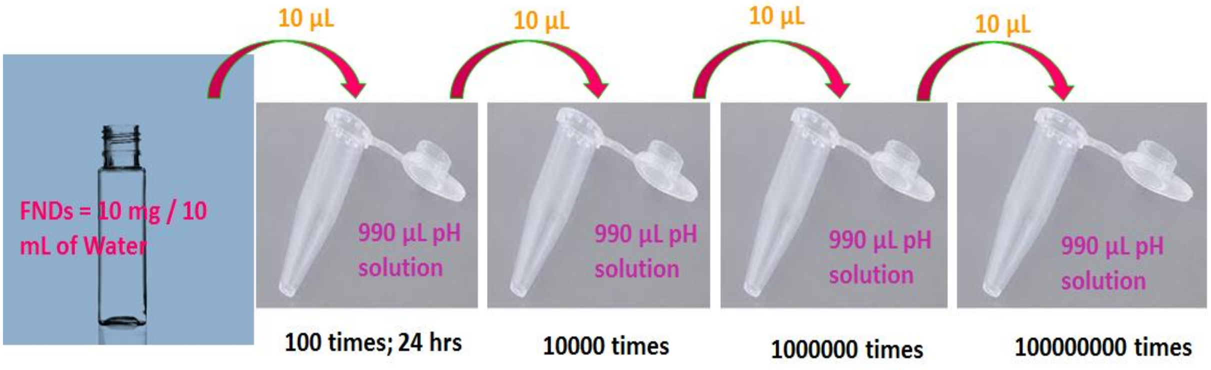

Figure S11. Representation of **ND-Cys** dispersion at pH 6 for SEM and TEM studies; before each dilution, the former solution was stirred to become homogeneous.

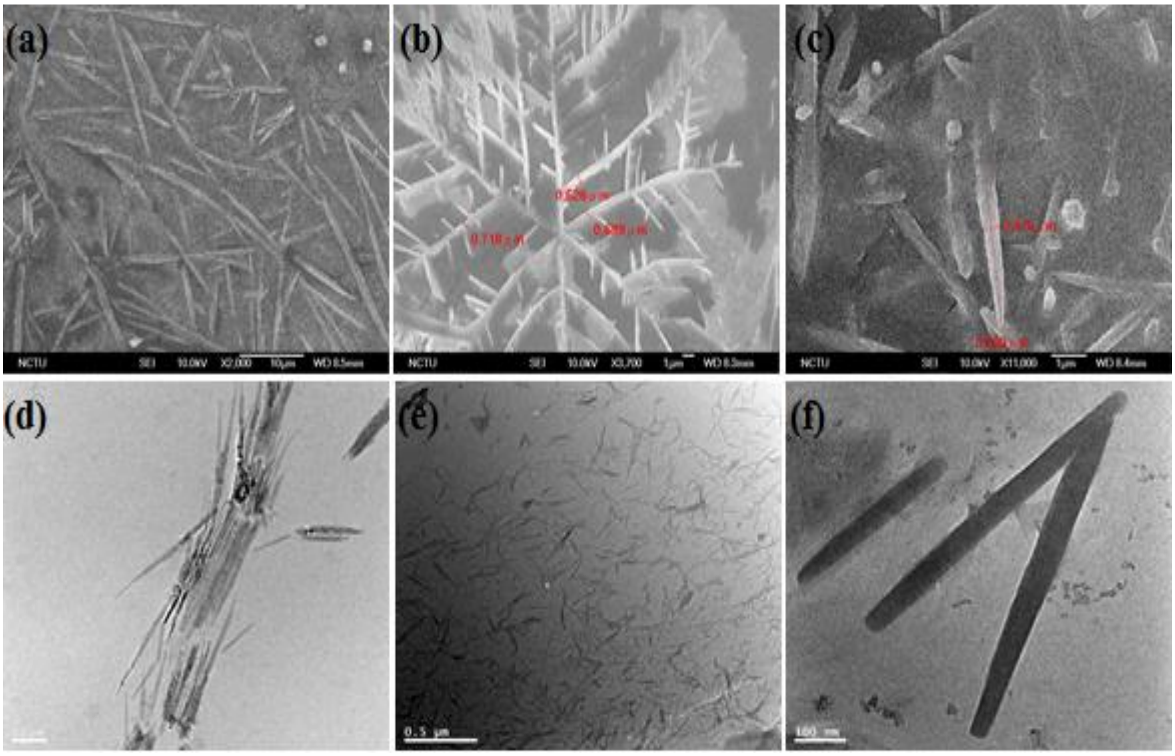

Figure S12. (a-c) SEM (10 pg/mL) and (d-f) TEM (1 fg/mL) of **ND-Cys** dispersion at pH 6 examined after 24 hours.

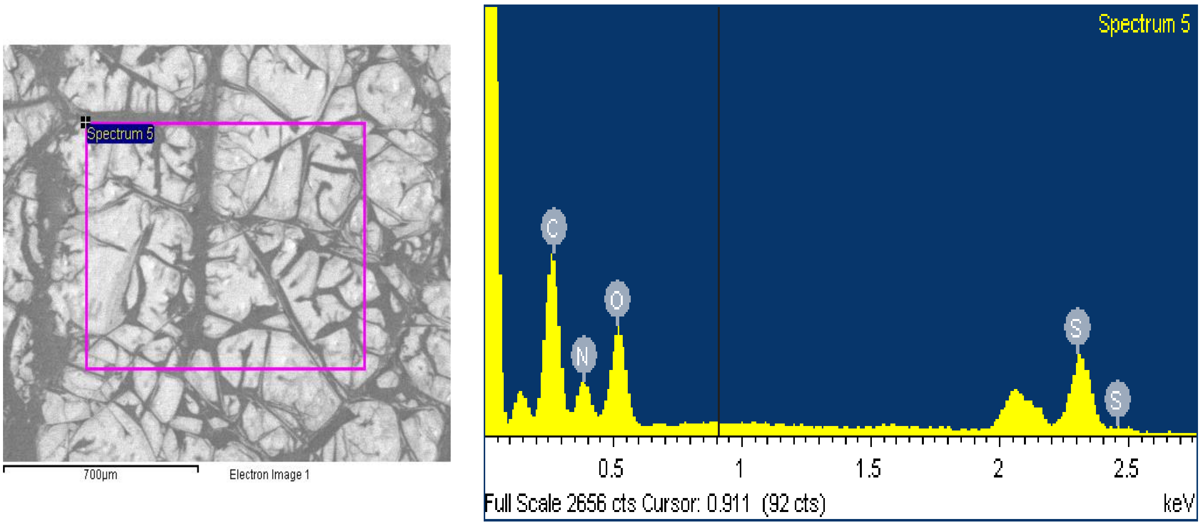

Figure S13. EDX spectrum of **ND-Cys** nanowires (G-DNWs).

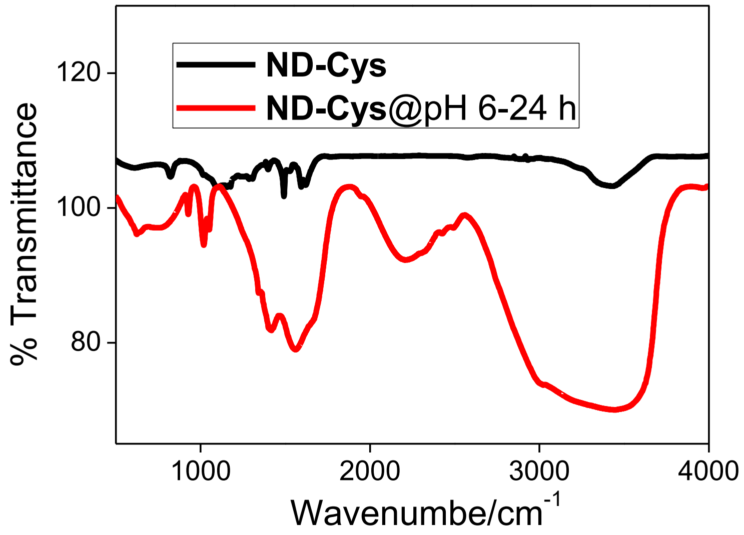

Figure S14. FTIR spectra of **ND-Cys** dispersion in pH 6 after 24 hours.

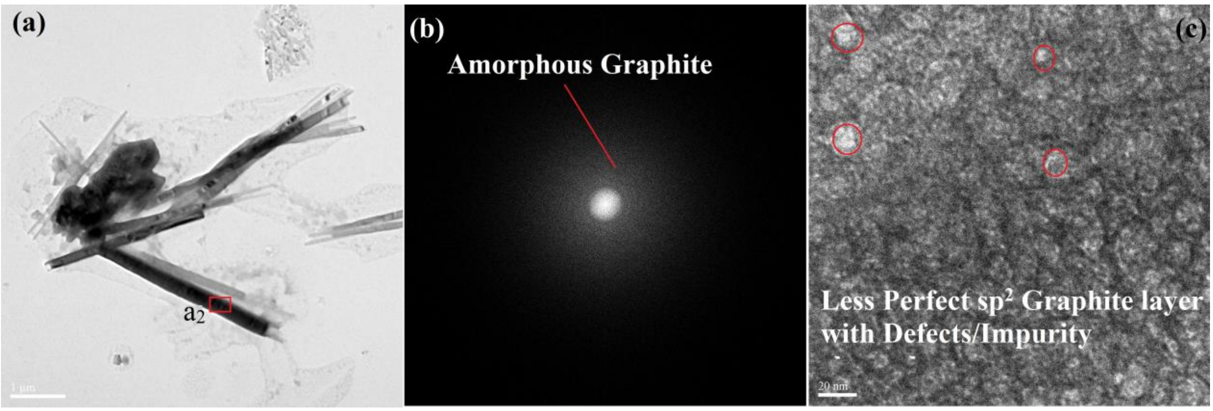

Figure S15. (a) HR-TEM image of G-DNWs (b) FT pattern of selected area $\mathbf{a_2}$ representing only amorphous graphite and (c) High magnification image of $\mathbf{a_2}$ region representing less perfect graphite layer along with defects or impurity outlined by red circles.

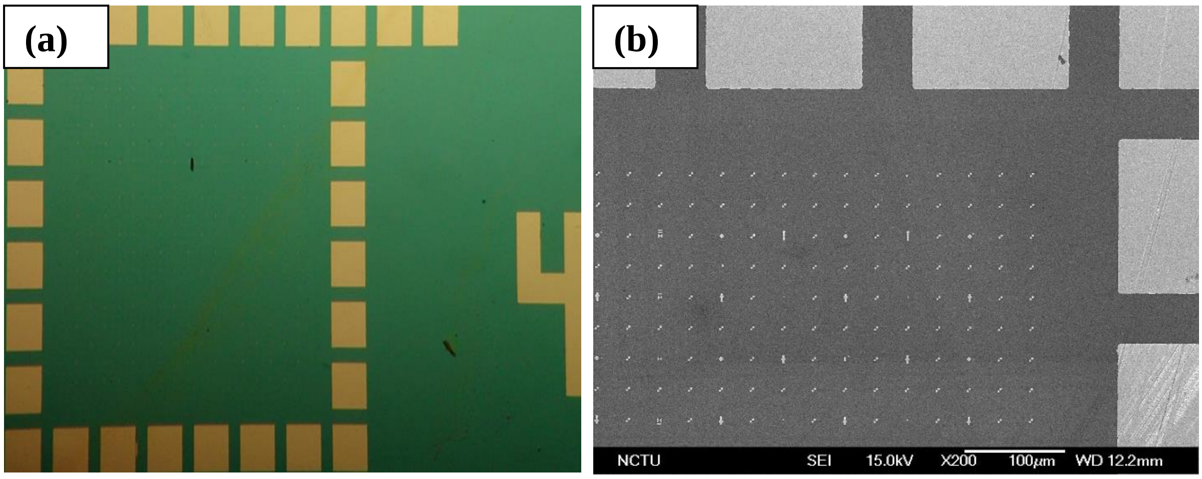

Figure S16. Images of (a) pad and (b) alignment mark.

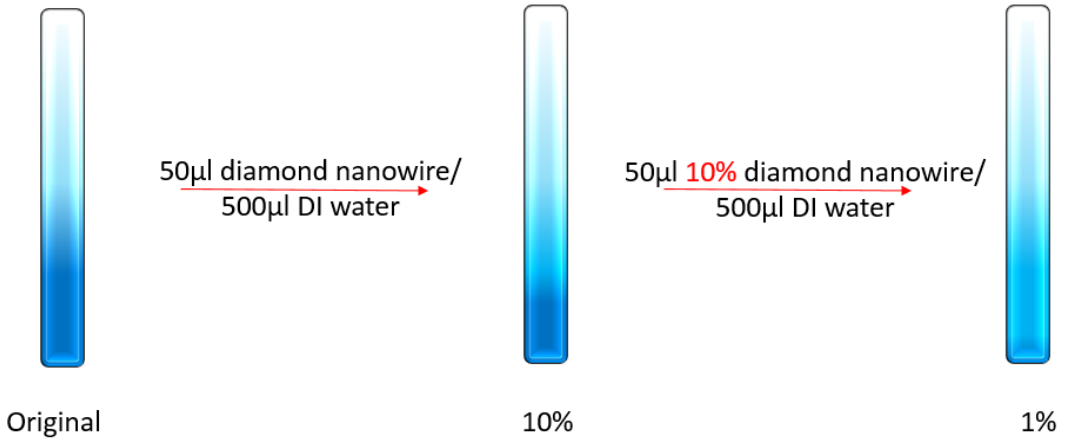

Figure S17. Representation of **ND-Cys** NW dispersion, final concentration is 100 fg/mL.

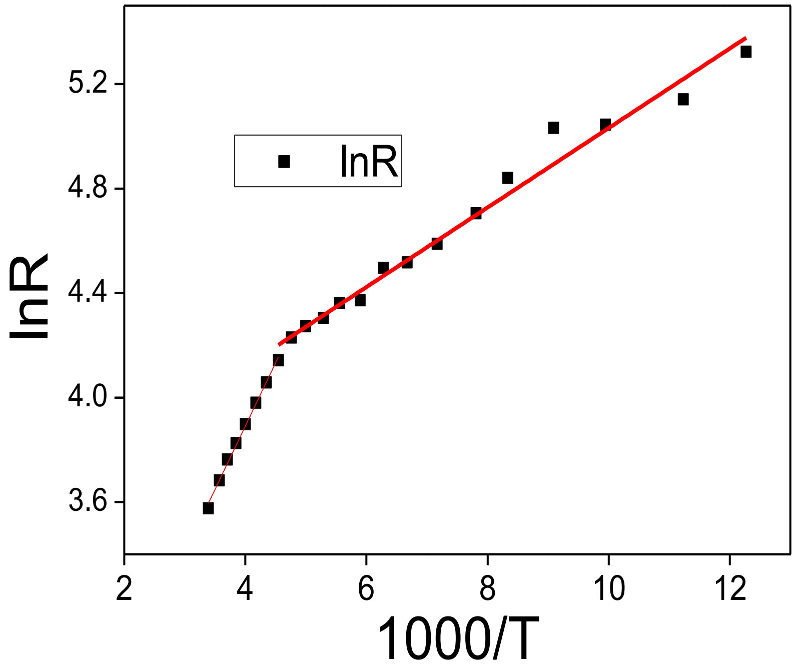

Figure S18. Activation energy (E_{a}) calculation from "lnR *Vs* 1000/T" plot based on Arrhenius equation $\mathbf{R = R_0}\ \boldsymbol{exp}^{\mathbf{(Ea/kT)}}$**.**

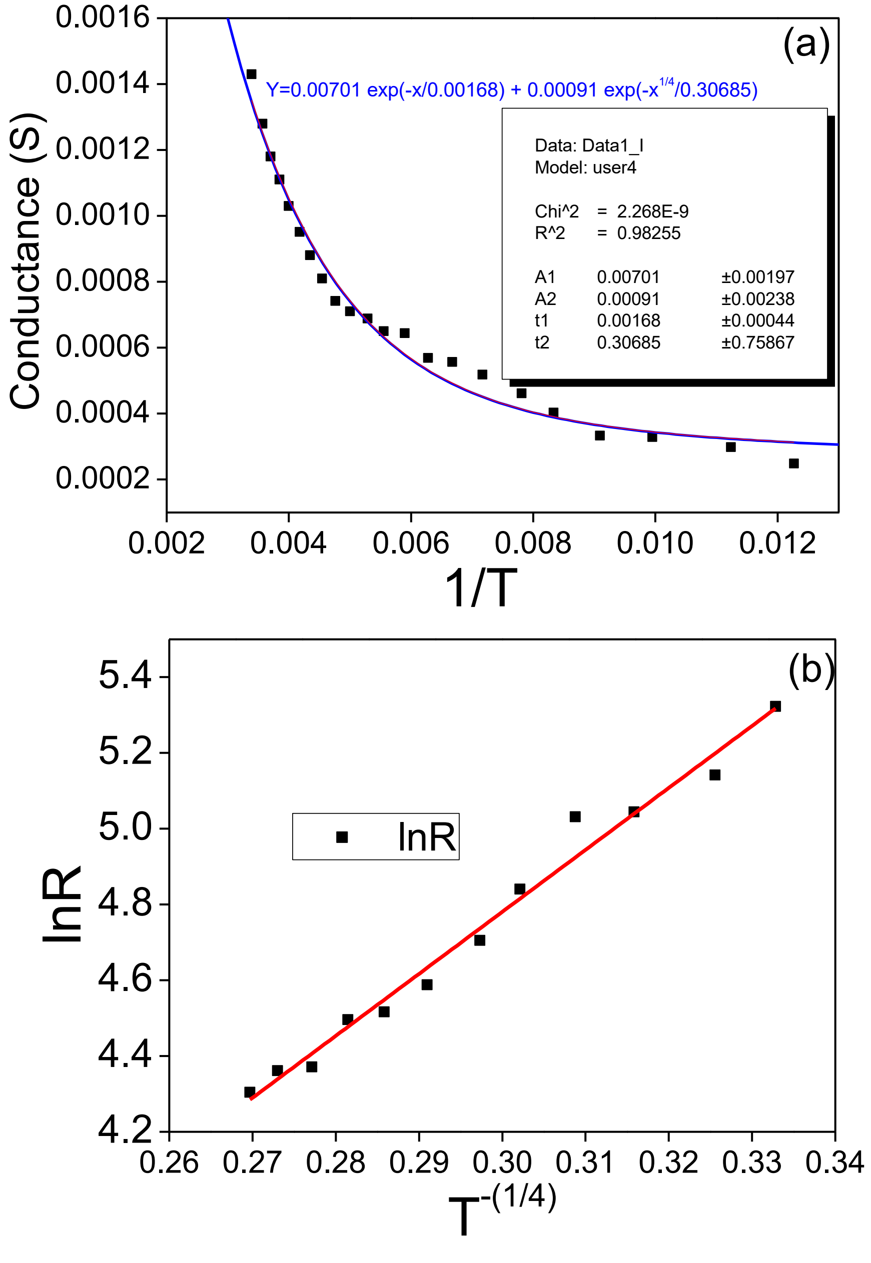

Figure S19. Plots of "Conductance *Vs* 1/T" and "lnR Vs $T^{(-1/4)}$" for evaluation of electron transport mechanism in **ND-Cys** NW conductivity by nonlinear and linear fittings, respectively.

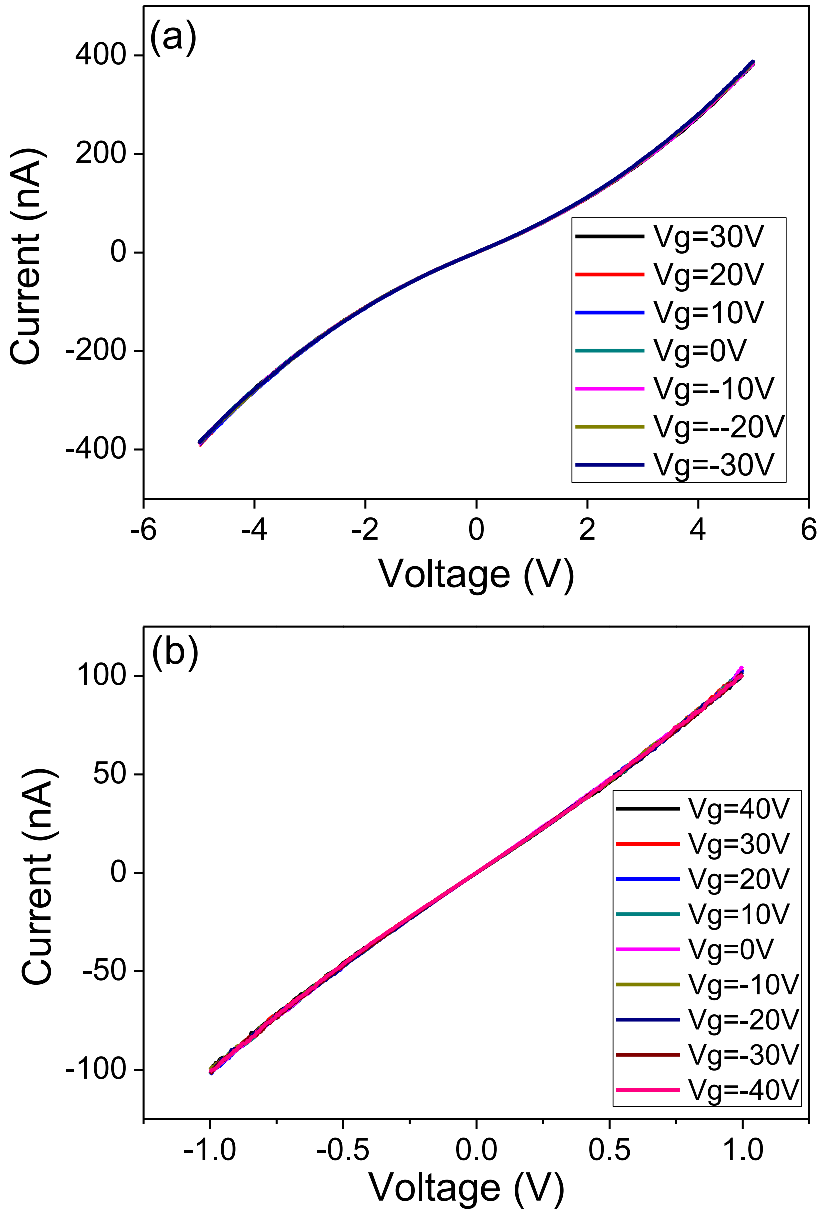

Figure S20. (a, b) MOSFET based conductivity measurements on L2 and L3 between -5 to 5 V and -1 to 1 V applied gate bias, respectively.
